# Synthesis of functionalized diazocines for application as building blocks in photo- and mechanoresponsive materials

**DOI:** 10.3762/bjoc.15.68

**Published:** 2019-03-20

**Authors:** Widukind Moormann, Daniel Langbehn, Rainer Herges

**Affiliations:** 1 Otto Diels Institute for Organic Chemistry, Christian-Albrechts-University, Otto-Hahn-Platz 4, 24118 Kiel, Germany

**Keywords:** bridged azobenzene, diazocine, mechanophor, oxidative C–C coupling, photochrome, reductive azo cyclization

## Abstract

Seven symmetrically 3,3’-substituted diazocines were synthesized. Functional groups include alcohol, azide, amine and vinyl groups, which are suitable for polymer synthesis. Upon irradiation at 385 and 530 nm the diazocines perform a reversible, pincer-type movement switching the 3,3’-distance between 6.1 Å (*cis*, stable isomer) and 8.2 Å (*trans*, metastable isomer). Key reactions in the synthesis are an oxidative C–C coupling of 2-nitrotoluenes (75–82% yield) and a reductive ring closure to form the diazocines (56–60% yield). The cyclization of the dinitro compound to the azo compound was improved in yield and reproducibility, by over-reduction to the hydrazine and reoxidation to the azo unit. In contrast to 3,3’- and 4,4’-diaminodiazocine, which have been implemented in macromolecules for conformation switching, our compounds exhibit improved photophysical properties (photostationary states, separation of absorption bands in the *cis* and *trans* configuration). Hence they are promising candidates as molecular switches in photo and mechanoresponsive macromolecules and other smart materials.

## Introduction

**Figure 1:**
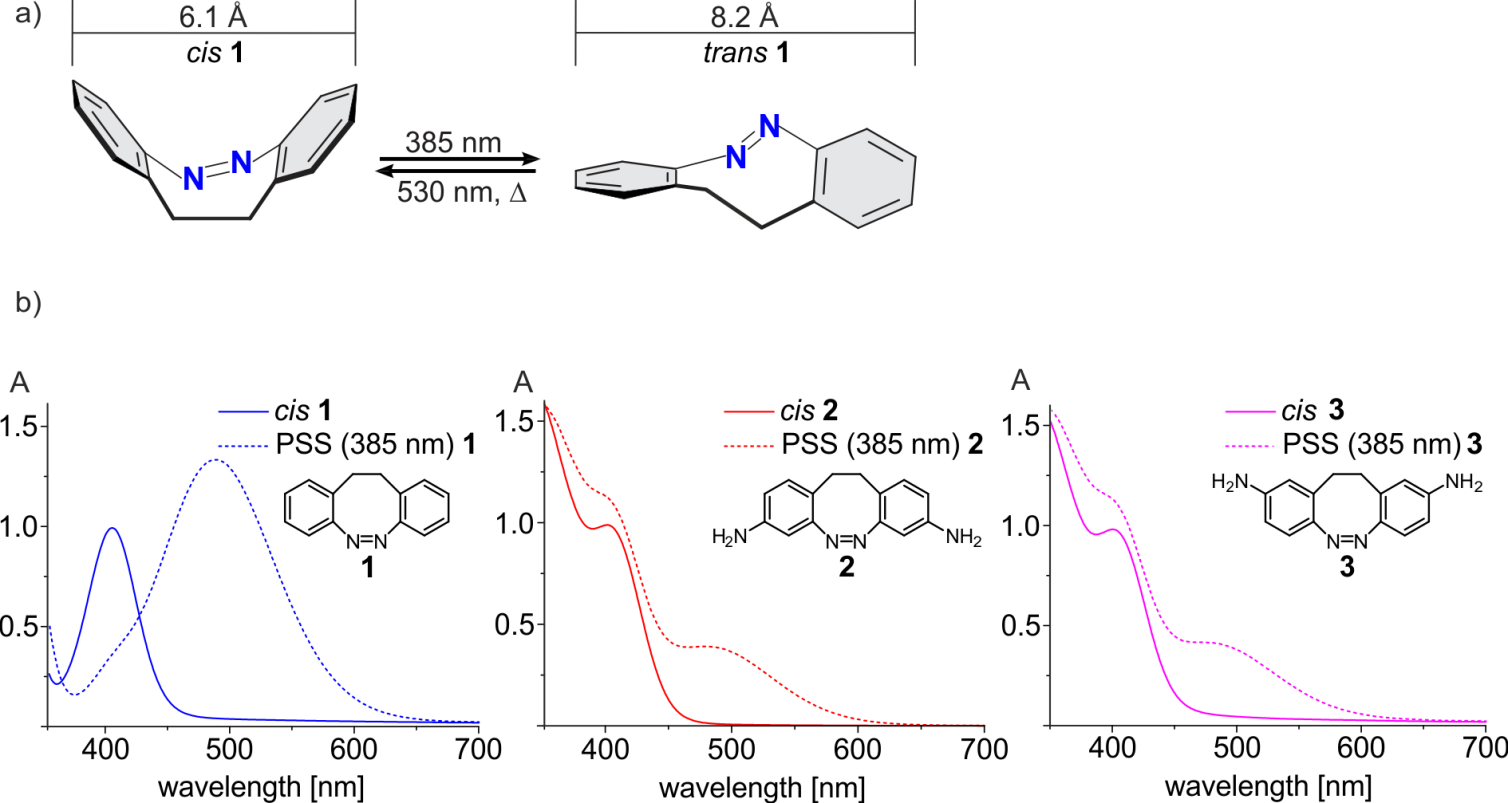
(a) Isomerization of parent diazocine **1**. Distances of carbon atoms *para* to the ethylene bridge were determined at the B3LYP/6-31g* level of DFT [23]. (b) UV–vis spectra of parent diazocine **1** (left), 3,3’-diaminodiazocine **2** (center) and 4,4’-diaminodiazocine **3** (right). Continuous lines: 100% *cis* and dashed lines: PSS (385 nm) at 298.15 K in acetonitrile.

The field of photoresponsive materials is of growing interest [1–3]. Several mechanophores such as azobenzene [4–8], diarylethene [9–13] and spiropyrans [14–18] have been investigated as photoswitchable building blocks. Bridged azobenzenes also known as diazocines exhibit excellent photochemical properties but applications are limited and suitably functionalized compounds are rare [19–23]. In contrary to azobenzenes, diazocines **1** are stable in their *cis* configuration. The bent *cis* isomer is less prone to π–π stacking which is known to reduce the switching efficiency (Figure 1a) [19,24]. The reverse stability of the *cis* and *trans* isomers in azobenzenes and diazocines should allow reciprocal applications in mechanoresponsive materials and in photopharmacology [25]. Another advantage of diazocines over azobenzenes is their switchability in the visible range (400 nm *cis* → *trans*, 530 nm *trans* → *cis*) preventing deterioration of the material or tissue damage by UV light [19]. Well separated absorption bands, high switching efficiency and high quantum yields are further advantages regarding their application as switches in photoresponsive materials [19,24,26]. In contrast to spiropyrans which have been frequently used as photoswitches in materials, diazocines are stable over several thousand switching cycles under air [19,24,26–27]. Notwithstanding their excellent properties, to date only 3,3’- and 4,4’- functionalized diazocine **2** and **3** have been implemented in polymers [19] and proteins [28]. Unfortunately, similar to azobenzenes, aminosubstitution at the phenyl rings reduces switching efficiency [29]. In contrast to the parent system, separation of absorption bands of the *cis* and *trans* isomer in **2** and **3** is poor. Upon irradiation of the corresponding *cis-*configured compounds at 385 nm only 30% of the *trans* isomer of 3,3’-diazocine **2** and 25% of the 4,4’-diazocine **3** are formed (Figure 1b). Applications of diazocines **2** and **3** are further hampered by the low yields of their synthesis [19–20].

**Figure 2:**
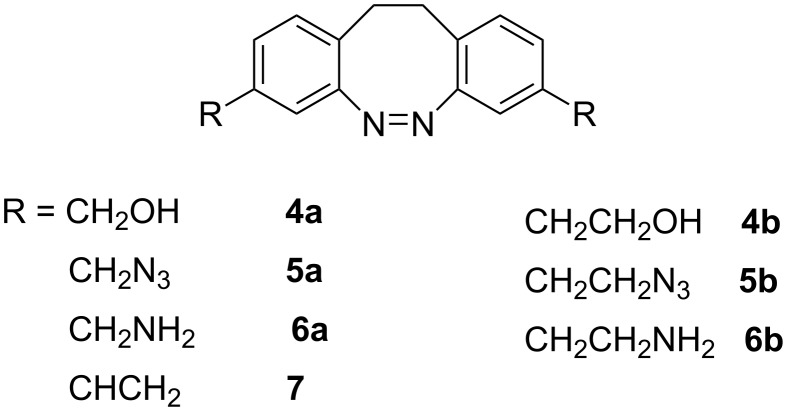
Synthesized target diazocines **4–7** for applications in responsive materials.

To decouple the electronic influence of the functional groups from the azo switching process and to improve yields of the azo cyclization step, we separated the functional groups from the aromatic system by one or two methylene groups and restricted substitution to the position *meta* with respect to the azo group (Figure 2) [19–20].

## Results and Discussion

**Scheme 1:**
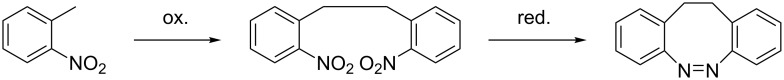
Key reactions in diazocine synthesis.

The synthesis of the targeted diazocines **4–7** is based on two key reactions, an oxidative C–C coupling of nitrotoluenes and the reductive ring closure of the dinitro compounds (Scheme 1). We recently improved the yield of the C–C coupling through addition of bromine as an oxidizing agent [27]. The reaction times thus are reduced to several minutes as compared to several hours in previous procedures using oxygen and the yields are increased from 65% to 95% in the parent system [30].

**Scheme 2:**
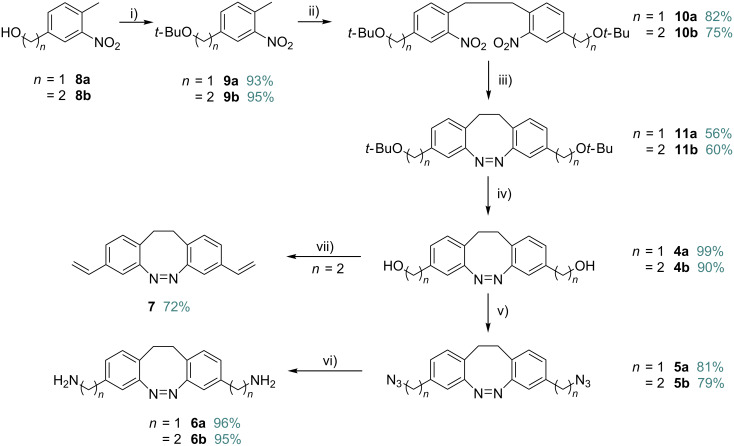
Syntheses of the functionalized diazocines **4–7**. Reaction conditions: i) Isobutylene, sulfuric acid, DCM; ii) *t*-BuOK, Br_2_, THF; iii) Zn, Ba(OH)_2_ H_2_O/EtOH, and CuCl_2_/O_2_, NaOH/MeOH; iv) Ti(Cl)_4_, DCM; v) ADMP, DBU, THF; vi) PPh_3_, H_2_O, THF; viii) TsCl, DMAP, TEA, DCM, and *t*-BuOK, THF.

In a recent work we observed that the reduction of 2,2′-dinitrodibenzyl is difficult to stop at the azo stage because further reduction to the hydrazine is faster than the preceding cyclization reaction [27]. The hydrazine is quite stable towards reduction to the diamine, and can easily be reoxidized to the azo compound using CuCl_2_/O_2_. The yields are higher and more reproducible using the above reduction/reoxidation scheme. Previously applied reducing agents include Ba(OH)_2_/Zn [27], glucose/NaOH [20], Pb/NEt_3_/HCOOH [22–23], or the Baeyer–Mills reaction via Zn/NH_4_Cl [25]. We chose the Ba(OH)_2_/Zn method because it provided superior yields even at larger scales. The syntheses of the functionalized diazocines **4–7** started with (4-methyl-3-nitrophenyl)methanol (**8a**) and (4-methyl-3-nitrophenyl)ethanol (**8b**). In a first step the hydroxy groups in **8a** and **8b** were protected as *tert*-butyl ethers (Scheme 2) to prevent oxidation in the following oxidative C–C coupling [31]. The *tert*-butyl ether was chosen as the protecting group because it is stable towards the oxidizing conditions of the C–C coupling reactions and the reducing conditions of the azo cyclization. Moreover, the *tert*-butyl group can be conveniently removed under acidic conditions. As described in [27] potassium butoxide is used as a non-nucleophilic base to remove the α-toluene protons of **9a** and **9b**. By addition of bromine as an oxidizing agent dimers **10a** and **10b** are formed, most probably through radical intermediates. Then the dinitro compounds **10a** and **10b** were reduced with Ba(OH)_2_/Zn to the hydrazine intermediates and subsequently oxidized with CuCl_2_ and air in a two-step reductive azo cyclization in a similar manner as described in [27]. After deprotection with TiCl_4_ the hydroxy-functionalized diazocines **4a** and **4b** were obtained [32]. The hydroxy groups in **4a** and **4b** were successfully converted into azides using 2-azido-1,3-dimethylimidazolinium hexafluorophosphate (ADMP) and DBU [33]. The synthesis was completed with a Staudinger reaction to obtain the amino-functionalized diazocines **6a** and **6b** [34]. Additionally, the diazocine **4b** was converted into the divinyldiazocine **7**. Towards this end, the hydroxy groups were tosylated, followed by elimination with potassium butoxide [35–36].

**Figure 3:**
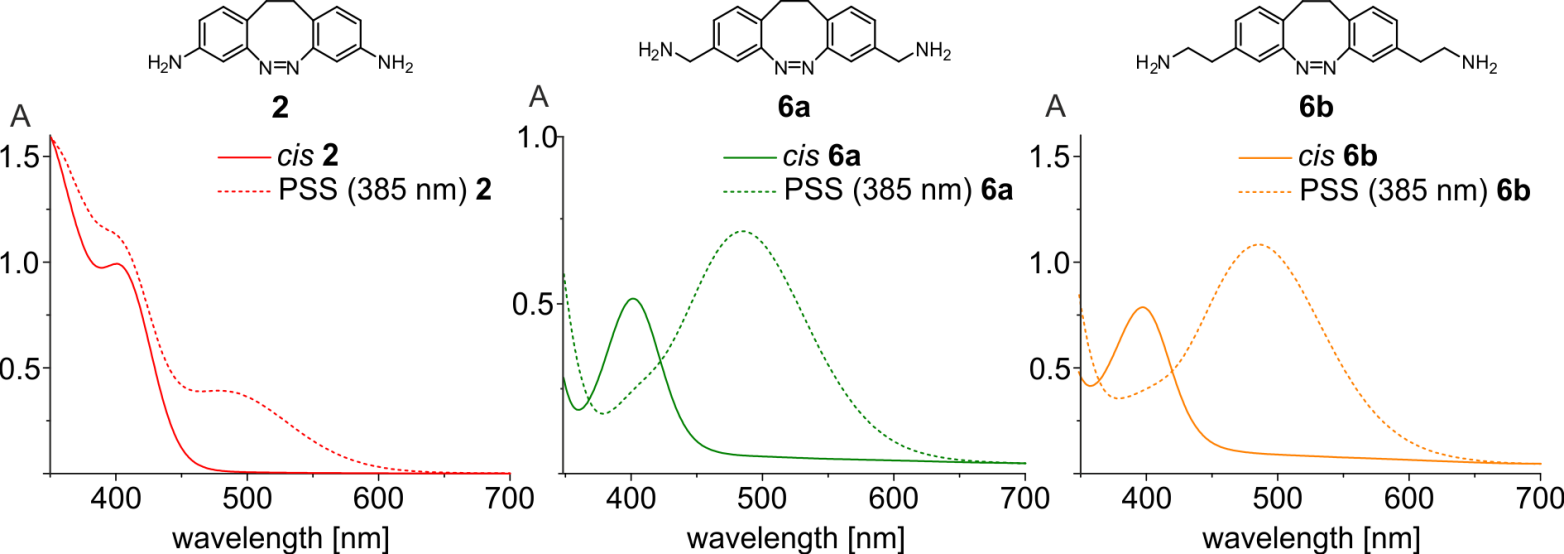
UV–vis spectra of 3,3’-diaminodiazocine **2** (left), 3,3’-di(aminomethyl)diazocine **6a** (center), and 3,3’-di(aminoethyl)diazocine **6b** (right). Continuous lines: 100% *cis* and dashed lines: PSS (385 nm) at 298.15 K in acetonitrile.

The photochemical and photophysical properties of compounds **4–7** were investigated by NMR and UV–vis spectroscopy and the results are listed in Table 1. Photostationary states (PSS) as well as half-lives (*t*_1/2_) were determined in acetonitrile at 300 K and 298.15 K, respectively. The (*cis* → *trans*) and (*trans* → *cis)* isomerization were achieved by irradiation into the appropriate n–π* bands at 385 and 530 nm. As a result of electronic decoupling the absorption bands are well separated and the photostationary states of diazocines **4–7** are considerably improved compared to 3,3’- (**2**) and 4,4’-diaminodiazocine **3** (Figure 3). The (*cis* → *trans*) isomerization of diazocines **4–7** was achieved after 2 min of irradiation at 385 nm in yields of 74–85%. All *trans*-diazocines were converted quantitatively to the *cis*-configuration either by thermal relaxation or by irradiation at 530 nm. In general, the (*trans* → *cis)* isomerization can be accomplished with wavelengths between 520 and 620 nm. The half-lives (*t*_1/2_) for the thermal relaxation (*trans* → *cis*) at 298.15 K of diazocines **4–7** in acetonitrile are between 10.2–16.7 h and thus are in the same order of magnitude as the parent system **1** (15.3 h). In comparison, the 3,3’-diaminodiazocine **2** has a much longer half-life (*t*_1/2_) of 24.5 h. The electronic decoupling of substituents in diazocines **4–7** has proven to retain the excellent photochemical properties in regard to PSS and half-life (*t*_1/2_) of the parent system **1**.

**Table 1:** Photostationary states (300 K), absorption maxima and half-lives (298.15 K), determined by ^1^H NMR and UV–vis spectroscopy in acetonitrile.

molecule	PSS (385 nm) [%] *trans*	PSS (530 nm) [%] *cis*	λ_max_ (*cis*) [nm]	λ_max_ (*trans*) [nm]	*t*_1/2_ (UV) [h] at 298.15 K

**1**	87	>99	402	486	15.3
**2**	30	>99	401	487	24.8
**3**	25	>99	400	475	20
**4a**	83	>99	404	485	11.4
**4b**	81	>99	405	487	16.7
**5a**	85	>99	402	484	11.2
**5b**	82	>99	405	489	14.0
**6a**	81	>99	405	488	14.7
**6b**	78	>99	400	485	10.1
**7**	74	>99	403	484	13.1
**11a**	82	>99	403	487	10.2
**11b**	81	>99	405	488	15.9

## Conclusion

Seven symmetrically substituted diazocines **4**–**7** were synthesized and characterized. Oxidative C–C coupling and reductive azo condensation proved to be reliable key steps in the synthesis of these substituted diazocines. The photophysical properties of compounds **4–7** were investigated by NMR and UV–vis experiments. The previously investigated 3,3’-diaminodiazocine **2** and 4,4’-diaminodiazocine **3** exhibited poor photostationary states (PSS (385 nm): 25–30% *trans*). The electronic decoupling of the azobenzene unit and the oxygen and nitrogen containing functional groups (OH, OR, N_3_, NH_2_) was achieved by insertion of one or two CH_2_ groups. Thereby, the switching efficiencies were increased by about a factor of two (PSS (385 nm): 74–85% *trans*), and thus are close to the parent system **1** (87%). Moreover, the yields of the two synthetic key steps, the oxidative C–C coupling and the azo cyclization have been improved. Diazocines **4**–**7** are easily accessible and valuable building blocks for the synthesis of photo- and mechanoresponsive polymers such as polyurethanes, polyesters, polyamides, polyureas and polyolefines.

## Supporting Information

File 1 Analytical equipment, experimental procedures, NMR and UV–vis spectra.
