# Supplementary material for: Synthesis of functionalized diazocines for application as building blocks in photo- and mechanoresponsive materials
Source: Beilstein J Org Chem. 2019 Mar 20;15:727–32. doi: 10.3762/bjoc.15.68 (PMC6444418; doi:10.3762/bjoc.15.68)
Supplement: File 1 — Analytical equipment, experimental procedures, NMR and UV–vis spectra. [file Beilstein_J_Org_Chem-15-727-s001.pdf]

BEILSTEIN JOURNAL OF ORGANIC CHEMISTRY

# Supporting Information

for

# Synthesis of functionalized diazocines for application as building blocks in photo- and mechanoresponsive materials

Widukind Moormann, Daniel Langbehn and Rainer Herges

*Beilstein J. Org. Chem.* **2019,** *15,* 727–732. doi:10.3762/bjoc.15.68

# Analytical equipment, experimental procedures, NMR and UV–vis spectra

License and Terms: This is a supporting information file under the terms of the Creative Commons Attribution License (http://creativecommons.org/licenses/by/4.0). Please note that the reuse, redistribution and reproduction in particular requires that the authors and source are credited.
The license is subject to the *Beilstein Journal of Organic Chemistry* terms and conditions: (https://www.beilstein-journals.org/bjoc)

# Table of contents

I. Analytical equipment· ........ S2

II. Syntheses· ........ S4

III. NMR spectra· ........ S17

IV. UV–vis spectra· ........ S30

V. Photochemical experiments· ........ S34

## I. Analytical equipment

### NMR spectroscopy

NMR spectra were measured in deuterated solvents (Deutero). To reference the NMR spectra the following solvent signals were used:

| solvent | degree of deuteration | $^{1}$H signal | $^{13}$C signal |
|---|---|---|---|
| acetone-$d_6$ | 99.8 % | 2.05 (quintet) | 29.84 (septet) |
| chloroform-$d_1$ | 99.8 % | 7.26 (s) | 77.16 (triplet) |
| acetonitril-$d_3$ | 99.8 % | 1.94 (quintet) | 118.26 (septet) |
| $D_2O$ | 99.8 % | 4.76 (s) | |

NMR measurements were performed with a Bruker DRX 500 ($^{1}$H-NMR: 500 MHz, $^{13}$C-NMR: 125 MHz) and a Bruker AV 600 ($^{1}$H-NMR: 600 MHz, $^{13}$C-NMR: 150 MHz).

### Melting point

Melting points were measured with a Melting Point B-560 (Büchi) in melting point tubes.

### Mass spectrometry

The high resolution (HR-EI) mass spectra were measured with an AccuTOF GCv 4G (Joel) with ionization energy of 70 eV. High resolution (APCI) mass spectra were measured with a Thermo Fischer Q Exactive Plus MS, Hybrid Quadrupol-Orbitrap and ASSY-APCI Probe-USI 4-4.7 kV by Dynamic Integrated Solutions.

### IR spectroscopy

Infrared spectra were measured on a Perkin-Elmer 1600 Series FTIR spectrometer with an A531-G Golden-Gate-Diamond-ATR-unit. Signals were abbreviated with w, m, s, vs for weak, medium, strong and very strong signal intensity.

### UV–vis spectroscopy

UV–vis spectra were recorded with a Lambda 14 UV/Vis spectrometer, Perkin–Elmer. Quartz cuvettes of 10 mm optical path length were used.

## Chromatography stationary phases

Flash column chromatography purfications were performed on a Biotage® type Isolera one with Biotage® Ultra cartridges (Biotage®, HP-Sphere™, particle diameter: 25 µm, cartridges sizes: 10 g, 25 g, 50 g and 100 g) were used. $R_f$ values were determined by thin layer chromatography on Polygram® SilG/$UV_{254}$ (Macherey Nagel, 0.2 mm particle size) and ALUGRAM® Xtra SIL G/$UV_{254}$ (Macherey Nagel, 0.2 mm particle size).

## Light sources

The irradiation of the samples was performed with LEDs with a wavelength of 385 and 530 nm from SAHLMANN PHOTOCHEMICAL SOLUTIONS with followed specifications:

- 385 nm: 12 x Nichia NCSU034A, FWHM = 9 nm, P(opt) = 12 x 340 mW,
- 530 nm: 16 x Luxeon LXML-PM01-0080, FWHM = 33 nm, P(opt) = 16 x 200 mW],

## II. Syntheses

### II.1. Synthesis of 4-(*tert*-butoxymethyl)-2-nitrotoluene (9a)

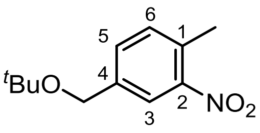


Under a $N_2$ atmosphere 4-hydroxymethyl-2-nitrotoluene (**8a**, 20.0 g, 120 mmol) was dissolved in 300 mL dry DCM, followed by the addition of isobutylene in DCM (300 mL, 251 mmol) and concentrated sulfuric acid (1.25 mL, 23.3 mmol). The reaction mixture was stirred for 16 h at room temperature and diluted with saturated sodium hydrogen carbonate (250 mL). The organic layer was separated and the aqueous layer was extracted with DCM (200 mL). The combined organic layers were washed with saturated sodium chloride solution, dried over magnesium sulfate and the solvent was removed in vacuo. The crude product was purified on silica flash column chromatography (cyclohexane/ethyl acetate, ethyl acetate 10% → 100%) to obtain a yellow oil (24.9 g, 111 mmol, 93%).

**$R_f$**: 0.60 (cyclohexane/ethyl acetate, 1:1).

**$^1$H-NMR** (500.1 MHz, $CDCl_3$, 300 K): $\delta$ = 7.96 (d, $^4J$ = 1.5 Hz, 1 H, *H*-3), 7.47 (dd, $^3J$ = 7.8 Hz, $^4J$ = 1.5 Hz, 1 H, *H*-5), 7.29 (d, $^3J$ = 7.8 Hz, 1 H, *H*-6), 4.47 (s, 2 H, $^t$BuO-C$H_2$), 2.57 (s, 3 H, C$H_3$), 1.30 (s, 9 H, *H*-$^t$Bu) ppm.

**$^{13}$C-NMR** (125.8 MHz, $CDCl_3$, 300 K): $\delta$ = 149.1 (*C*-2), 139.5 (*C*-4), 132.6 (*C*-6), 132.1 (*C*-1), 131.7 (*C*-5), 123.3 (*C*-3), 73.9 (*C*-($CH_3)_3$), 62.8 (*C*$H_2$), 27.6 (C-(*C*$H_3)_3$), 20.2 (*C*$H_3$) ppm.

**IR** (ATR): $\tilde{\nu}$ = 2974 (m), 2933 (w), 2870 (w), 1526 (s), 1453 (w), 1389 (w), 1346 (s), 1235 (w), 1192 (s), 1089 (m), 1025 (w), 883 (m), 814 (s), 755 (w), 680 (w) cm$^{-1}$.

**MS** (EI, 70 eV): m/z (%) = 150 (100), 208 (16), 104 (6).

**MS** (EI, HR, 70 eV): $C_{12}H_{17}NO_3$, m/z = calc.: 223.1208, found: 223.1211.

**II.2. Synthesis of 1,2-bis(4-(*tert*-butoxymethyl)-2-nitrophenyl)ethane (10a)**

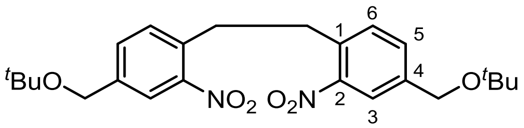


Under a $N_2$ atmosphere 4-(*tert*-butoxymethyl)-2-nitrophenyl)ethane (**9a**, 1.00 g, 4.48 mmol) was dissolved in 90 mL dry THF, cooled to −5 °C, followed by potassium butoxide addition (653 mg, 5.82 mmol). The reaction mixture was stirred for 30 s before the addition of bromine (274 µL, 5.38 mmol). After further stirring for 10 min the reaction mixture was added to 1 L ice. The precipitate was filtered and recrystallized from ethanol to obtain colourless crystals (816 mg, 1.84 mmol, 82%).

**melting point**: 131 °C.

$\mathbf{R}_f$: 0.80 (cyclohexane/ethylacetate, 1:1).

**$^1$H-NMR** (500.1 MHz, $CDCl_3$, 300 K): $\delta$ = 7.95 (d, $^4J$ = 1.5 Hz, 2 H, *H*-3), 7.51 (dd, $^3J$ = 7.8 Hz, $^4J$ = 1.5 Hz, 2 H, *H*-5), 7.38 (d, $^3J$ = 7.8 Hz, 2 H, *H*-6), 4.49 (s, 4 H, $^t$BuO-C$H_2$), 3.19 (s, 4 H, $C_2H_4$), 1.31 (s, 18 H, *H*-$^t$Bu) ppm.

**$^{13}$C-NMR** (125.8 MHz, $CDCl_3$, 300 K): δ = 149.1 (*C*-2), 140.2 (*C*-4), 134.7 (*C*-1), 132.4 (*C*-6), 131.9 (*C*-5), 123.3 (*C*-3), 74.8 (*C*-$CH_3$), 62.7 ($^t$BuO-*C*$H_2$), 34.2 ($C_2H_4$), 27.6 (C-($CH_3$)$_3$) ppm.

**IR** (ATR): ῦ = 2977 (m), 1569 (w), 1520 (vs), 1390 (m), 1362 (s), 1344 (s), 1195 (vs), 1120 (m), 1063 (s), 1025(m), 888 (s), 833 (s), 814 (s), 750 (m), 677 (m) $cm^{-1}$.

**MS** (APCI): m/z (%) = 483 (100) [M+K], 467 (12) [M+Na].

**MS** (APCI): m/z (%) = [$C_{24}H_{32}O_6N_2{}^{39}K$], m/z = calc.: 483.1892, found: 483.1890.

= [$C_{24}H_{32}O_6N_2{}^{23}Na$], m/z = calc.: 476.2153, found: 476.2152.

**II.3. Synthesis of (*Z*)-3,8-bis(*tert*-butoxymethyl)-11,12-dihydrodibenzo[*c*,*g*][1,2]diazocine (11a)**

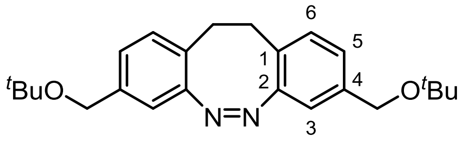


A mixture of 1,2-bis(4-(*tert*-butoxymethyl)-2-nitrophenyl)ethane (**10a**, 193 mg, 434 µmol), $Ba(OH)_2$ $8H_2O$ (411 mg, 1.30 mmol) and zinc powder (454 mg, 6.94 mmol) were dissolved in an ethanol/water mixture (60 mL, 2:1) and stirred for 5 h under reflux. After filtration trough Celite and evaporation of the solvent under reduced pressure, the crude product was dissolved in 0.1 M methanolic NaOH solution (150 mL), $CuCl_2$ (5.00 mg, 37.2 µmol) was added, and air was led trough the solution for 4 h. The reaction mixture was neutralized with 2 M HCl and the aqueous layer was extracted with DCM (3 × 75 mL). The combined organic layers were washed with a saturated sodium chloride solution and dried over magnesium sulfate. The solvent was evaporated in vacuo and the crude product was purified by flash column chromatography (cyclohexane/ethyl acetate, ethyl acetate 10% → 100%) to afford the product as a yellow solid (93.1 mg, 245 µmol, 56%).

**melting point**: 128 °C.

**R$_f$**: 0.59 (cyclohexane/ethyl acetate, 1:1).

**$^1$H-NMR** (500.1 MHz, $CDCl_3$, 300 K): $\delta$ = 6.99 (dd, $^3J$ = 7.8 Hz, $^4J$ = 1.7 Hz, 2 H, *H*-5), 6.92 (d, $^3J$ = 7.8 Hz, 2 H, *H*-6), 6.84 (d, $^4J$ = 1.7 Hz, 2 H, *H*-3), 4.35 (d, $^4J$ = 3.6 Hz, 4 H, C$H_2$), 3.01-2.65 (m, 4 H, $C_2H_4$), 1.24 (s, 9 H, *H*-$^t$Bu) ppm.

**$^{13}$C-NMR** (125.8 MHz, $CDCl_3$, 300 K): $\delta$ = 155.2 (*C*-2), 138.7 (*C*-4), 129.5 (*C*-6), 126.6 (*C*-1), 125.8 (*C*-5), 117.9 (*C*-3), 73.5 (*C*-$(CH_3)_3$), 63.4 ($CH_2$), 31.5 ($C_2H_4$), 27.6 (C-$(CH_3)_3$) ppm.

**IR** (ATR): $\tilde{\nu}$ = 2974 (s), 2872 (w), 1458 (w), 1388 (m), 1361 (s), 1192 (s), 1061 (s), 1022 (m), 902 (m), 830 (s), 814 (m), 771 (m) 746 (w) $cm^{-1}$.

**MS** (EI, 70 eV): m/z (%) = 380 (37), 323 (1), 307 (7), 279 (100), 193 (72).

**MS** (EI, HR, 70 eV): $C_{24}H_{32}N_2O_2$, m/z = calc.: 380.2463, found: 380.2463.

**II.4. Synthesis of (*Z*)-(11,12-dihydrodibenzo[*c*,*g*][1,2]diazocine-3,8-diyl)dimethanol (4a)**

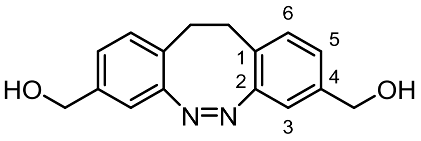

Under a $N_2$ atmosphere (*Z*)-3,8-bis(*tert*-butoxymethyl)-11,12-dihydrodibenzo[*c*,*g*][1,2]diazocine (**11a**, 100 mg, 260 µmol) was dissolved in 1 mL dry DCM, cooled to 0 °C and $TiCl_4$ (90.0 µL, 789 µmol) was added. After 1 min 20 mL saturated potassium carbonate solution were added. The solution was acidified with 1 M hydrochloric acid solution and extracted with DCM. The combined organic layers were washed with saturated sodium chloride solution 50 mL, dried over magnesium sulfate and the solvent was removed in vacuo. The crude product was purified on silica flash column chromatography (cyclohexane/ethyl acetate) to obtain a yellow solid. (70.0 mg, 260 µmol, 99%).

**melting point:** 133 °C.

**R$_f$:** 0.11 (cyclohexane/ethylacetate, 1:1).

**$^1$H-NMR** (500.1 MHz, $CDCl_3$, 300 K): $\delta$ = 7.00 (dd, $^3J$ = 8.0 Hz, $^4J$ = 1.6 Hz, 2 H, *H*-5), 6.96 (d, $^3J$ = 7.9 Hz, 2 H, *H*-6), 6.83 (d, $^4J$ = 1.1 Hz, 2 H, *H*-3), 4.56 (s, 4 H, C*H*$_2$-OH, 2.95 (s, 2 H, $C_2H_4$), 2.77 (s, 2 H, $C_2H_4$) ppm.

**$^{13}$C-NMR** (125.8 MHz, $CDCl_3$, 300 K): δ = 155.4 (*C*-2), 139.6 (*C*-1), 129.9 (*C*-6), 127.3 (*C*-4), 125.6 (*C*-5), 117.4 (*C*-3), 64.5 (*C*H$_2$-OH), 31.4 ($C_2H_4$) ppm.

**IR** (ATR): ῦ = 3258 (m), 2939(w), 2884 (w), 1525 (w), 1459 (w), 1405 (m), 1353 (w), 1276 (w), 1225 (w), 1092 (w), 1019 (vs), 996 (vs), 889 (m), 833 (s), 818 (s), 751 (s), 724 (m), 701 (m), 620 (m) $cm^{-1}$.

**MS** (EI, 70 eV): m/z (%) = 268 (100), 236 (83), 207 (56).

**MS** (EI, HR, 70 eV): $C_{16}H_{16}N_2O_2$, m/z = calc.: 268.1212, found: 268.1199.

**II.5. Synthesis of (*Z*)-3,8-bis(azidomethyl)-11,12-dihydrodibenzo[*c*,*g*][1,2]diazocine (5a)**

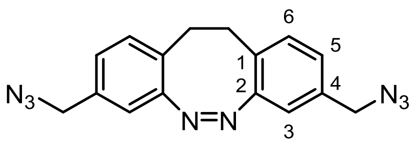


Under a $N_2$ atmosphere (*Z*)-(11,12-dihydrodibenzo[*c*,*g*][1,2]diazocine-3,8-diyl)dimethanol (**4a**, 1.00 g, 3.73 mmol) was dissolved in dry THF (20 mL). 2-Azido-1,3-dimethylimidazolinium hexafluorophosphate (2.65 g, 9.29 mmol)) and DBU (1.71 g, 11.2 mmol) were added slowly. After stirring for 30 min at room temperature the reaction mixture was diluted with saturated potassium carbonate solution. The organic layer was separated and the aqueous layer was extracted with DCM (3 × 50 mL), washed with saturated sodium chloride solution and dried over magnesium sulfate. The solvent was evaporated in vacuo and the crude product was then purified by flash column chromatography (cyclohexane/ethyl acetate, ethyl acetate 10% → 100%) to obtain a yellow solid (956 mg, 3.00 mmol, 81%).

**melting point**: 62 °C.

$\mathbf{R}_f$: 0.50 (cyclohexane/ethyl acetate, 1:1).

**$^1$H-NMR** (600.1 MHz, $CDCl_3$, 300 K): $\delta$ = 7.01-6.95 (m, 4 H, *H*-5, *H*-6), 6.76 (d, $^4J$ = 1.2 Hz, 2 H, *H*-3), 4.22 (m, 4 H, $CH_2$), 3.04-2.72 (m, 4 H, $C_2H_4$) ppm.

**$^{13}$C-NMR** (150.9, $CDCl_3$, 300 K): $\delta$ = 155.6 (*C*-4), 134.2 (*C*-2), 130.3 (*C*-6), 128.2 (*C*-1), 126.7 (*C*-5), 118.3 (*C*-3), 53.9 ($CH_2$), 31.3 ($C_2H_4$) ppm.

**IR** (ATR): $\tilde{\upsilon}$ = 2931 (w), 2093 (s), 1451 (w), 1338 (m), 1247 (s), 1092 (w), 874 (m), 826 (s), 807 (m), 750 (m), 630 (w), 611 (w) $cm^{-1}$.

**MS** (EI, 70 eV): m/z (%) = 318 (15), 262 (4), 248 (13), 233 (15), 178 (100).

**MS** (EI, HR, 70 eV): $C_{16}H_{14}N_8$, m/z = calc.: 318.1341, found: 318.1339.

## II.6. Synthesis of (*Z*)-(11,12-dihydrodibenzo[1,2]diazocin-3,8-diyl)dimethanamine (6a)

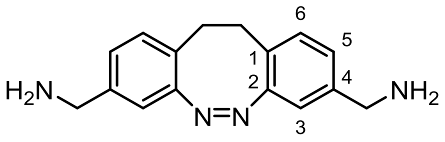


(*Z*)-(11,12-dihydrodibenzo[*c*,*g*][1,2]diazocine-3,8-diyl)dimethanol (**5a**, 78 mg, 245 µmol) and triphenylphosphine (141 mg, 540 µmol) were dissolved in dry THF. After the addition of water (88 µL, 490 µmol) the reaction mixture was stirred for 1 h at room temperature and afterwards diluted with water. The solution was acidified with 1 M hydrochloric acid solution and washed with DCM (3 × 50 mL). The aqueous solution was neutralized with 1 M sodium hydroxide solution and extracted at pH > 7 with DCM. The combined organic layers were washed with saturated sodium chloride solution, dried over magnesium sulfate and the solvent was removed in vacuo to obtain a yellow solid. (62.9 mg, 236 µmol, 96%).

**melting point:** 118 °C.

**$R_f$:** 0.01 (cyclohexane/ethylacetate, 1:1).

**$^1$H-NMR** (500.1 MHz, $CDCl_3$, 300 K): $\delta$ = 6.97 (dd, $^3J$ = 7.7 Hz, $^4J$ = 2.1 Hz, 2 H, *H*-5), 6.95 (d, $^3J$ = 7.7 Hz, 2 H, *H*-6), 6.81 (d, $^4J$ = 1.1 Hz, 2 H, *H*-3), 3.78 (s, 4 H, $CH_2$-$NH_2$), 3.02-2.70 (m, 4 H, $C_2H_4$) ppm.

**$^{13}$C-NMR** (125.8 MHz, $CDCl_3$, 300 K): δ = 155.4 (*C*-2), 141.9 (*C*-4), 129.8 (*C*-6), 126.4 (*C*-1), 125.8 (*C*-5), 117.5 (*C*-3), 45.8 ($CH_2$-$NH_2$), 31.4($C_2H_4$) ppm.

**IR** (ATR): ṽ = 2871 (m), 1556 (s), 1455 (vs), 1372 (s), 1297 (vs), 911 (m), 811 (vs), 695 (m) $cm^{-1}$.

**MS** (EI, 70 eV): m/z (%) = 266 (59), 237 (100) 220 (82).

**MS** (EI, HR, 70 eV): $C_{16}H_{18}N_4$, m/z = calc.: 266.1532, found: 266.1543.

## II.7. Synthesis of 4-(2-(*tert*-butoxy)ethyl)-2-nitrotoluene (9b)

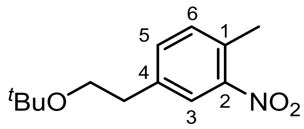


Under a $N_2$ atmoshere 4-(2-(hydroxy)ethyl)-2-nitrotoluene (**8b**, 2.63 g, 14.5 mmol) was dissolved in 150 mL dry DCM, followed by the addition of isobutylene in DCM (150 mL, 126 mmol) and concentrated sulfuric acid (700 µL, 13.1 mmol). The reaction mixture was stirred for 15 h at room temperature and afterwards diluted with saturated sodium hydrogen carbonate (50 mL). The organic layer was separated and the aqueous layer was extracted with DCM (3 × 50 mL). The combined organic layers were washed with saturated sodium chloride solution, dried over magnesium sulfate and the solvent was removed in vacuo. The crude product was purified on silica flash column chromatography (cyclohexane/ethyl acetate, ethyl acetate 10% → 100%) to obtain a yellow oil (3.28 g, 13.6 mmol, 95%).

**R*f***: 0.63 (cyclohexane/ethyl acetate, 1:1).

**$^1$H-NMR** (500.1 MHz, $CDCl_3$, 300 K): $\delta$ = 7.87 (d, $^4J$ = 1.7 Hz, 1 H, *H*-3), 7.37 (dd, $^3J$ = 7.8 Hz, $^4J$ = 1.7 Hz, 1 H, *H*-5), 7.23 (d, $^3J$ = 7.8 Hz, 1 H, *H*-6), 3.56 (t, $^3J$ = 6.8 Hz, 2 H, $^t$BuO-C$H_2$), 2.85 (t, $^3J$ = 6.8 Hz, 2 H, $^t$BuO-CH2-C*H*2), 3.22 (s, 3 H, C$H_3$), 1.15 (s, 9 H, *H*-$^t$Bu) ppm.

**$^{13}$C-NMR** (125.8 MHz, $CDCl_3$, 300 K): $\delta$ = 149.0 (*C*-2), 139.1 (*C*-4), 133.9 (*C*-5), 132.5 (*C*-6), 131.1 (*C*-1), 125.0 (*C*-3), 73.0 (*C*-($CH_3)_3$), 62.0 ($^t$BuO-*C*$H_2$), 36.3 ($^t$BuO-$CH_2$-*C*$H_2$), 27.5 (C-(*C*$H_3)_3$), 20.1 (*C*$H_3$) ppm.

**IR** (ATR): $\tilde{\nu}$ = 2973 (m), 2933 (m), 2870 (w), 1526 (s), 1498 (w), 1453 (w), 1362 (m), 1345 (m), 1193 (m), 1079 (m), 1022 (w), 940 (w), 889 (w), 807 (m), 738 (m), 679 (w) cm$^{-1}$.

**MS** (EI, 70 eV): m/z (%) = 238 (8), 207 (52), 182 (42), 151 (100), 134 (88).

**MS** (EI, HR, 70 eV): $C_{13}H_{20}NO_2$, m/z = calc.: 238.1443, found: 238.1445.

## II.8. Synthesis of 1,2-bis(4-(2-(*tert*-butoxy)ethyl)-2-nitrophenyl)ethane (10b)

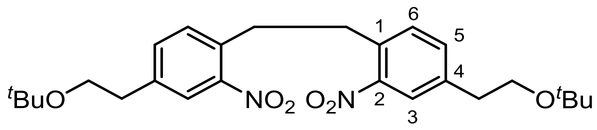


Under a $N_2$ atmosphere 4-(*tert*-butoxyethyl)-2-nitrophenyl)ethane (**9b**, 4.00 g, 16.9 mmol) was dissolved in 80 mL dry THF, cooled to −5 °C, followed by potassium butoxide addition (2.65 g, 23.6 mmol). The reaction mixture was stirred for 30 s before the addition of bromine (1.12 mL, 21.9 mmol). After further stirring for 20 min the reaction mixture was added to 1 L ice. The precipitate was filtered and recrystallized from ethanol to obtain colourless crystals (2.97 g, 6.29 mmol, 75%).

**melting point**: 72 °C.

**$R_f$:** 0.51 (cyclohexane/ethylacetate, 3:1).

**$^1$H-NMR** (500.1 MHz, $CDCl_3$, 300 K): $\delta$ = 7.85 (d, $^4J$ = 1.7 Hz, 2 H, *H*-3), 7.40 (dd, $^3J$ = 7.8 Hz, $^4J$ = 1.7 Hz, 2 H, *H*-5), 7.30 (d, $^3J$ = 7.8 Hz, 2 H, *H*-6), 3.57 (t, $^3J$ = 6.8 Hz, 2 H, ($^t$BuO-C$H_2$), 3.19 (s, 4 H, $C_2H_4$), 2.87 (t, $^3J$ = 6.7 Hz, 2 H, ($^t$BuO-CH$_2$-C$H_2$), 1.16 (s, 18 H, *H*-$^t$Bu) ppm.

**$^{13}$C-NMR** (125.8 MHz, $CDCl_3$, 300 K): δ = 149.2 (*C*-2), 140.0 (*C*-1), 134.7 (*C*-4), 134.1 (*C*-5), 132.1 (*C*-6), 125.09 (*C*-3), 73.1 (*C*-$CH_3$), 62.0 ($^t$BuO-*C*$H_2$), 36.6 ($^t$BuO-CH$_2$-*C*H$_2$) 34.2 ($C_2H_4$), 27.3 (C-($CH_3$)$_3$) ppm.

**IR** (ATR): ῦ = 2974 (m), 2872 (w), 1566 (w), 1526 (vs), (1463 (w), 1392 (w), 1362 (s), 1348 (s), 1266 (m), 1196 (s), 1079 (s), 1029 (w), 939 (w), 894 (w), 843 (m), 830 (w), 806 (m), 766 (w), 736 (w), 677 (w) $cm^{-1}$.

**MS** (APCI): m/z (%) = 511 (100) [M+K].

**MS** (HR-APCI): m/z (%) = [$C_{26}H_{36}O_6N_2{}^{39}K$], m/z = calc.: 511.2205, found: 511.2204.

**II.9. Synthesis of (*Z*)-3,8-bis(2-(*tert*-butoxy)ethyl)-11,12-dihydrodibenzo[*c*,*g*][1,2]diazocine (11b)**

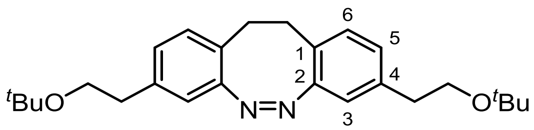


To a solution of 1,2-bis(4-(2-(*tert*-butoxy)ethyl)-2-nitrophenyl)ethane (**10b**, 900 mg, 1.90 mmol) in 105 mL of ethanol, $Ba(OH)_2 \cdot 8H_2O$ (1.80 g, 5.71 mmol) in 50 mL of $H_2O$ and zinc powder (1.99 g, 30.5 mmol) were added, and the mixture was stirred for 5 h under reflux. The reaction mixture was filtered through Celite, and the solvent was removed under reduced pressure. The crude product was dissolved in DCM, filtered through Celite, and the solvent was removed under reduced pressure. The crude product was dissolved in 120 mL 0.1 M methanolic sodium hydroxide solution, $CuCl_2$ (20 mg, 149 µmol) was added and air was led through the solution until completion of the reaction. The reaction mixture was neutralized with 6 M HCl solution. After the addition of saturated sodium bicarbonate solution the aqueous layers were extracted with DCM. The combined organic layers were dried over $MgSO_4$ and the solvent was reduced under reduced pressure. The crude product was purified by flash column chromatography (cyclohexane/ethyl acetate, 3:1) to obtain a yellow oil (466 mg, 1.14 mmol, 60%).

**$R_f$ :** 0.44 (cyclohexane/ethyl acetate, 3:1).

**$^1$H-NMR** (500.1 MHz, $CDCl_3$, 300 K): $\delta$ = 6.88 (m, 4 H, *H*-5, *H*-6), 6.67 (s, 2 H, *H*-3), 3.45 (t, $^3J$ = 7.1 Hz, 4 H, $^t$BuO-C$H_2$-$CH_2$), 2.82 (m, 4 H, $C_2H_4$), 2.70 (t, $^3J$ = 7.1 Hz, 4 H, $^t$BuO-$CH_2$-C$H_2$), 1.08 (s, 18 H, *H*-$^t$Bu) ppm.

**$^{13}$C-NMR** (125.8 MHz, $CDCl_3$, 300 K): δ = 155.2 (*C*-2), 138.2 (*C*-4), 129.4 (*C*-6), 127.8 (*C*-1), 125.7 (*C*-5), 119.4 (*C*-3), 72.8 (*C*-($CH_3$)$_3$), 62.5 ($^t$BuO-*C*$H_2$), 36.7 ($^t$BuO-$CH_2$-*C*$H_2$), 31.4 (*C*$_2$H$_4$), 27.4 (C-(*C*$H_3$)$_3$) ppm.

**IR** (ATR): ṽ = 2972 (m), 2866 (w), 1611 (w), 1566 (w), 1491 (w), 1390 (m), 1362 (s), 1233 (w), 1194 (s), 1078 (vs), 1020 (w), 939 (w), 879 (m), 811 (m), 740 (m), 628 (w) $cm^{-1}$.

**MS** (EI, 70 eV): m/z (%) = 408 (40), 321 (18), 293 (82), 279 (28), 264 (16), 208 (100).

**MS** (EI, HR, 70 eV): $C_{26}H_{36}N_2O_2$, m/z = calc.: 408.2777, found: 408.2771.

**II.10. Synthesis of (*Z*)-2,2'-(11,12-dihydrodibenzo[*c*,*g*][1,2]diazocine-3,8-diyl)bis(ethan-1-ol) (4b)**

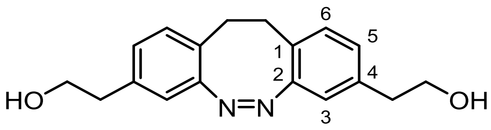


Under a $N_2$ atmosphere (*Z*)-3,8-bis(2-(*tert*-butoxy)ethyl)-11,12-dihydrodibenzo[*c*,*g*][1,2]diazocine (**11b**, 340 mg, 833 µmol) was dissolved in 3 mL dry DCM, cooled to 0 °C and $TiCl_4$ (90.0 µL, 789 µmol) was added. After 30 s 20 mL saturated potassium carbonate solution were added. The solution was acidified with 1 M hydrochloric acid solution and extracted with DCM. The combined organic layers were washed with saturated sodium chloride solution (50 mL), dried over magnesium sulfate and the solvent was removed in vacuo. The crude product was purified on silica flash column chromatography (cyclohexane/ethyl acetate, 1:1) to obtain a yellow solid. (221 mg, 746 µmol, 90%).

**melting point**: 94 °C.

**$R_f$:** 0.09 (cyclohexane/ethyl acetate, 1:1).

**$^1$H-NMR** (500.1 MHz, $CDCl_3$, 300 K): $\delta$ = 6.90 (d, $^3J$ = 7.8 Hz, 2 H, *H*-6), 6.96 (dd, $^3J$ = 7.5 Hz, $^4J$ = 1.6 Hz 2 H, *H*-5), 6.67 (d, $^4J$ = 1.6 Hz, 2 H, *H*-3), 3.72 (t, $^3J$ = 6.2 Hz, 4 H, HO-C$H_2$), 2.82 (s, 4 H, $C_2H_4$), 2.73 (t, $^3J$ = 6.4 Hz, 4 H, HO-$CH_2$-C$H_2$ ) ppm.

**$^{13}$C-NMR** (125.8 MHz, $CDCl_3$, 300 K): δ = 155.7 (*C*-2), 137.7 (*C*-4), 130.0 (*C*-6), 126.4 (*C*-1), 127.8 (*C*-5), 118.9 (*C*-3), 63.7 (HO-*C*$H_2$), 38.8 (HO-$CH_2$-*C*$H_2$), 31.4 ($C_2H_4$) ppm.

**IR** (ATR): ṽ = 3294 (m), 2918 (m), 1402 (m), 1329 (m), 1068 (s), 1030 (vs), 886 (w), 860 (w), 797 (s), 618 (s) cm$^{-1}$.

**MS** (EI, 70 eV): m/z (%) = 296 (32), 265 (10), 237 (100), 219 (32), 204 (55).

**MS** (EI, HR, 70 eV): $C_{18}H_{18}N_2O_2$, m/z = calc.: 296.1525, found: 296.1509.

**II.11. Synthesis of (*Z*)-3,8-bis(2-azidoethyl)-11,12-dihydrodibenzo[*c*,*g*][1,2]diazocine (5b)**

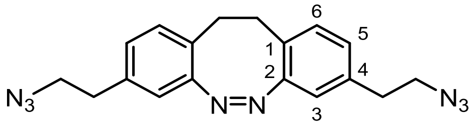


Under a $N_2$ atmosphere (*Z*)-2,2'-(11,12-dihydrodibenzo[*c*,*g*][1,2]diazocine-3,8-diyl)bis(ethan-1-ol) (**4b**, 132 mg, 445 µmol) was dissolved in 2 mL dry THF. 2-Azido-1,3-dimethylimidazolinium hexafluorophosphate (316 mg, 1.11 mmol) and DBU (199 µL, 1.34 mmol) were added, the reaction was stirred for 2 h at room temperature and stopped by the addition of 20 mL saturated potassium solution. After extraction with DCM the combined organic layers were washed with saturated sodium chloride solution, dried over magnesium sulfate and the solvent was removed in vacuo. The crude product was purified on silica flash column chromatography (cyclohexane/ethyl acetate, 1:1) to obtain a yellow oil. (121 mg, 350 µmol, 79%).

**$R_f$:** 0.8 (cyclohexane/ethyl acetate, 1:1).

**$^1$H-NMR** (500.1 MHz, $CDCl_3$, 300 K): $\delta$ = 6.93 (d, $^3J$ = 7.8 Hz, 2 H, *H*-6), 6.87 (dd, $^3J$ = 7.8 Hz, $^4J$ = 1.7 Hz 2 H, *H*-5), 6.70 (d, $^4J$ = 1.6 Hz, 2 H, *H*-3), 3.41 (t, $^3J$ = 5.8 Hz, 4 H, $N_3$-C$H_2$), 2.94 (s, 4 H, $C_2H_4$), 2.78 (t, $^3J$ = 7.1 Hz, 4 H, $N_3$-$CH_2$-C$H_2$ ) ppm.

**$^{13}$C-NMR** (125.8 MHz, $CDCl_3$, 300 K): δ = 155.5 (*C*-2), 136.8 (*C*-4), 129.6 (*C*-6), 127.5 (*C*-1), 126.6 (*C*-5), 119.0 (*C*-3), 52.1 ($N_3$-*C*$H_2$), 34.8 ($N_3$-$CH_2$-*C*$H_2$), 31.4 ($C_2H_4$) ppm.

**IR** (ATR): ṽ = 2928 (w), 2087 (vs), 1685 (w), 1610 (w), 1566 (m), 1492 (m), 1485 (m), 1437 (m), 1346 (m), 1252 (s), 1137 (w), 1091 (w), 1043 (w), 979 (w), 948 (w), 887 (m), 809 (m) $cm^{-1}$.

**MS** (EI, 70 eV): m/z (%) = 346 (34), 262 (18), 234 (22), 218 (30), 202 (67), 191 (100).

**MS** (EI, HR, 70 eV): $C_{18}H_{18}N_8$, m/z = calc.: 346.1654, found: 346.1650.

**II.12. Synthesis of (*Z*)-2,2'-(11,12-dihydrodibenzo[*c*,*g*][1,2]diazocine-3,8-diyl)bis(ethan-1-amine) (6b)**

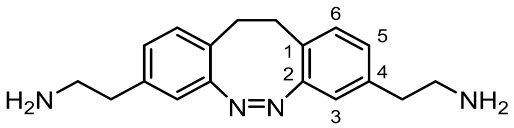


(*Z*)-3,8-Bis(2-azidoethyl)-11,12-dihydrodibenzo[*c*,*g*][1,2]diazocine (**5b**, 68 mg, 196 µmol) and triphenylphosphine (113 mg, 431 µmol) were dissolved in dry THF. After the addition of water (71.0 µL, 392 µmol) the reaction mixture was stirred for 4 h at room temperature and afterwards diluted with water. The solution was acidified with 1 M hydrochloric acid solution and washed with DCM (3 × 50 mL). The aqueous solution was neutralized with 1 M sodium hydroxide solution and extracted at pH > 7 with DCM. The combined organic layers were washed with saturated sodium chloride solution, dried over magnesium sulfate and the solvent was removed in vacuo to obtain a yellow solid. (55.0 mg, 187 µmol, 95%).

**$R_f$:** 0.01 (cyclohexane/ethyl acetate, 1:1).

**$^1$H-NMR** (500.1 MHz, $D_2O$, 300 K): $\delta$ = 7.11 (d, $^3J$ = 7.9 Hz, 2 H, *H*-6), 7.03 (dd, $^3J$ = 7.9 Hz, $^4J$ = 1.7 Hz, 2 H, *H*-5), 6.83 (d, $^4J$ = 1.6 Hz, 2 H, *H*-3), 3.17 (t, $^3J$ = 6.2 Hz, 4 H, $H_2N$-C$H_2$), 2.87 (s, 4 H, $C_2H_4$), 2.86 (m, 4 H, $H_2N$-$CH_2$-C$H_2$ ) ppm.

**$^{13}$C-NMR** (125.8 MHz, $D_2O$, 300 K): δ = 154.1 (*C*-2), 136.7 (*C*-4), 130.7 (*C*-6), 128.5 (*C*-1), 128.1 (*C*-5), 119.0 (*C*-3), 40.1 ($N_3$-*C*$H_2$), 32.1 ($N_3$-$CH_2$-*C*$H_2$), 30.5 ($C_2H_4$) ppm.

**IR** (ATR): ṽ = 2944 (w), 1777 (w), 1671 (s), 1520 (w), 1469 (w), 1434 (w), 1135 (vs), 962 (m), 839 (m), 812 (s), 797 (s), 750 (w), 723 (s), 704 (s) $cm^{-1}$.

**MS** (EI, 70 eV): m/z (%) = 294 (1), 265 (100), 236 (11), 220 (35), 205 (54).

**MS** (EI, HR, 70 eV): $C_{18}H_{22}N_4$, m/z = calc.: 294.1845, found: 294.1839.

**II.13. Synthesis of (*Z*)-3,8-divinyl-11,12-dihydrodibenzo[*c*,*g*][1,2]diazocine (7)**

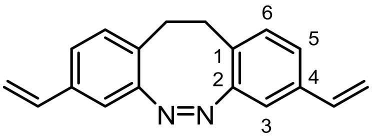

Under a $N_2$ atmosphere tosylchloride (325 mg, 1.71 mmol) and DMAP (27 mg, 223 µmol) were dissolved in 4 mL dry DCM and TEA (414 mL, 2.97 mmol) was added. (*Z*)-2,2'-(11,12-Dihydrodibenzo[*c*,*g*][1,2]diazocine-3,8-diyl)bis(ethan-1-ol) (**4b**, 220 mg, 742 µmol) was dissolved in 7 mL dry DCM and added dropwise over a period of 10 min. The reaction mixture was stirred for 5 h at room temperature, followed by the addition of saturated sodium carbonate solution. After extraction with DCM the combined organic layers were washed with water and saturated sodium chloride solution and dried over magnesium sulfate. The solvent was removed in vacuo and the crude product was purified on silica flash column chromatography (cyclohexane/ethyl acetate, 1:1). The isolated product from step one was dissolved under $N_2$ atmosphere in dry THF and potassium butoxide (151 mg, 1.35 mmol) was dissolved in dry THF and added dropwise at 0 °C. The reaction mixture was stirred at 0 °C for 30 min and diluted in ethyl acetate and water. After separation, the aqueous phase was extracted with ethyl acetate twice, washed with saturated sodium chloride solution twice, and dried over magnesium sulfate. The solvent was removed under reduced pressure and the residue purified by silica flash column chromatography (cyclohexane/ethyl acetate, 1:1) to obtain yellow crystals. (139 mg, 534 µmol, 72%).

**melting point**: 98 °C.

**R*f***: 0.65 (cyclohexane/ethyl acetate, 2:1).

**$^1$H-NMR** (500.1 MHz, $CDCl_3$, 300 K): $\delta$ = 7.06 (dd, $^3J$ = 7.8 Hz, $^4J$ = 1.7 Hz 2 H, *H*-5), 6.94 (d, $^3J$ = 7.9 Hz, 2 H, *H*-6), 6.87 (d, $^4J$ = 1.7 Hz, 2 H, *H*-3), 6.59 (dd, $^2J$ = 17.6 Hz, $^3J$ = 10.9 Hz, 2 H, C*H*-$CH_2$), 5.66 (dd, $^3J$ = 17.6 Hz, $^4J$ = 0.7 Hz, 2 H, CH-C$H_{2\ trans}$), 5.21 (dd, $^3J$ = 10.9 Hz, $^4J$ = 0.6 Hz, 2 H, CH-C$H_{2\ cis}$), 2.86 (m, 4 H, $C_2H_4$) ppm.

**$^{13}$C-NMR** (125.8 MHz, $CDCl_3$, 300 K): δ = 155.4 (*C*-2), 136.2 (*C*-4), 135.8 (*C*H-$CH_2$), 129.9 (*C*-6), 127.6 (*C*-1), 124.9 (*C*-5), 116.7 (*C*-3), 114.5 (CH-*C*$H_2$), 31.5 ($C_2H_4$), ppm.

**IR** (ATR): ṽ = 2021 (w), 1836 (w), 1556 (w), 1487 (w), 1434 (w), 1267 (w), 1093 (w), 997 (m), 986 (m), 956 (m), 914 (vs), 900 (s), 884 (m), 819 (s), 749 (m), 707 (w) $cm^{-1}$.

**MS** (EI, 70 eV): m/z (%) = 260 (78), 215 (53), 202 (100).

**MS** (EI, HR, 70 eV): $C_{18}H_{16}N_2$, m/z = calc.: 260.1313, found: 260.1317.

## III. NMR spectra

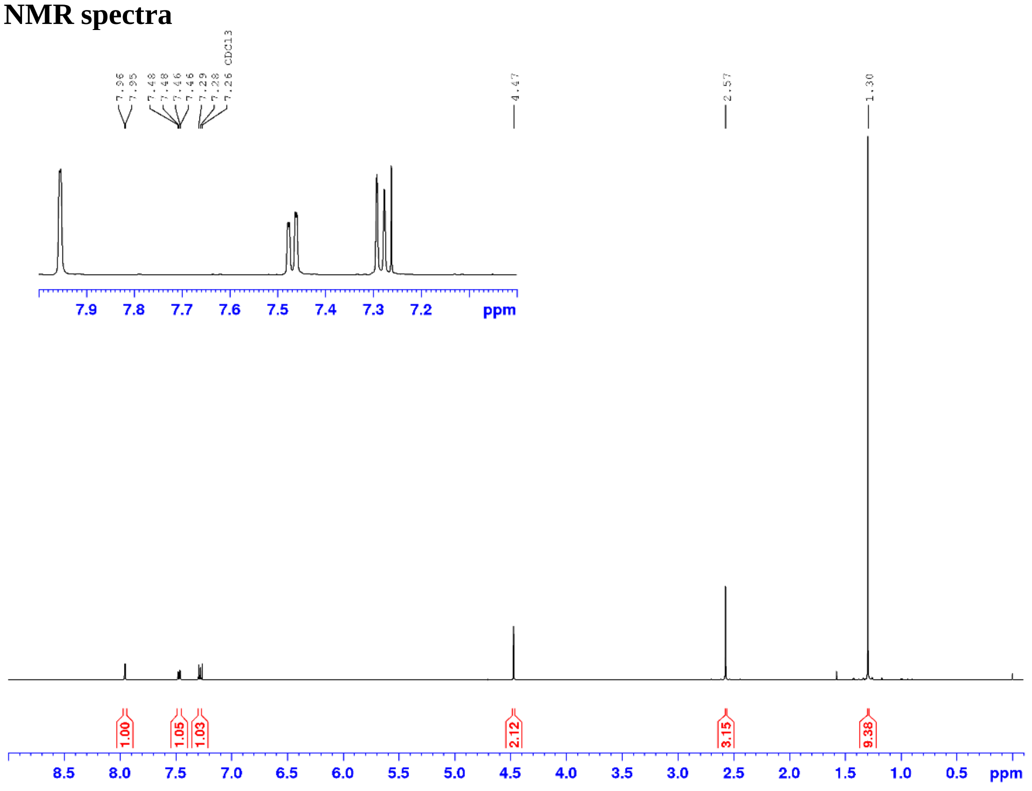

**Figure S1**: $^{1}$H-NMR spectrum of compound **9a** measured in deuterated chloroform.

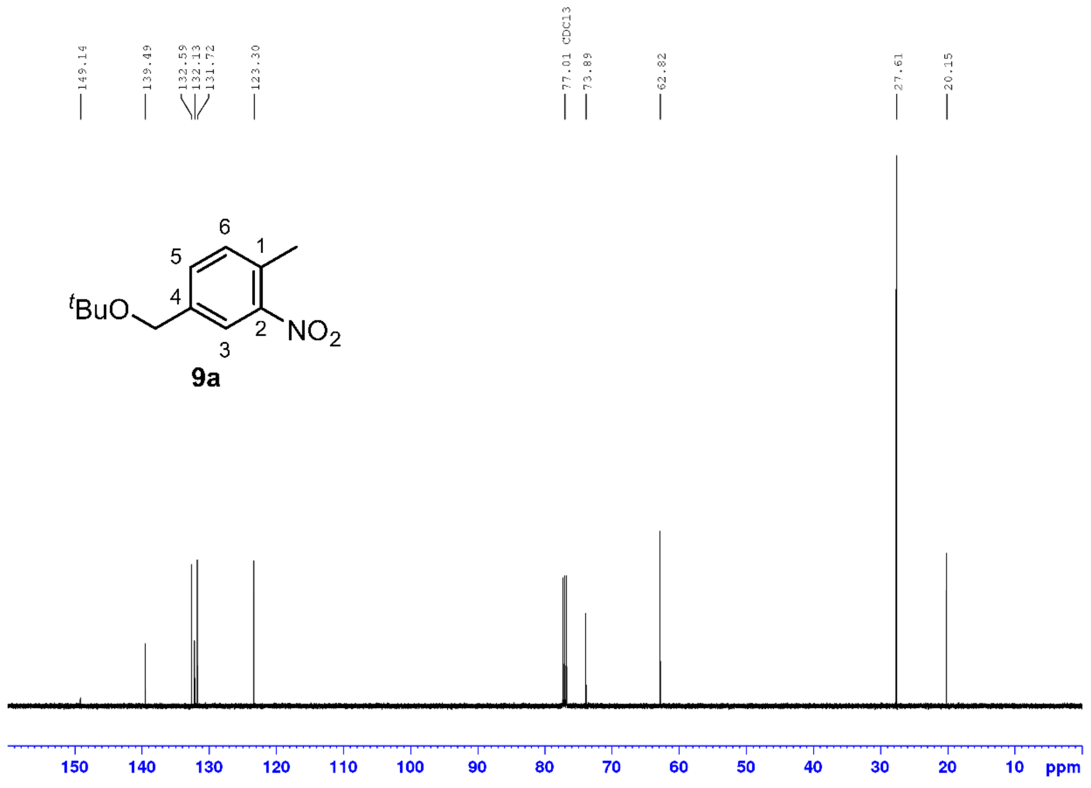

**Figure S2**: $^{13}$C-NMR spectrum of compound **9a** measured in deuterated chloroform.

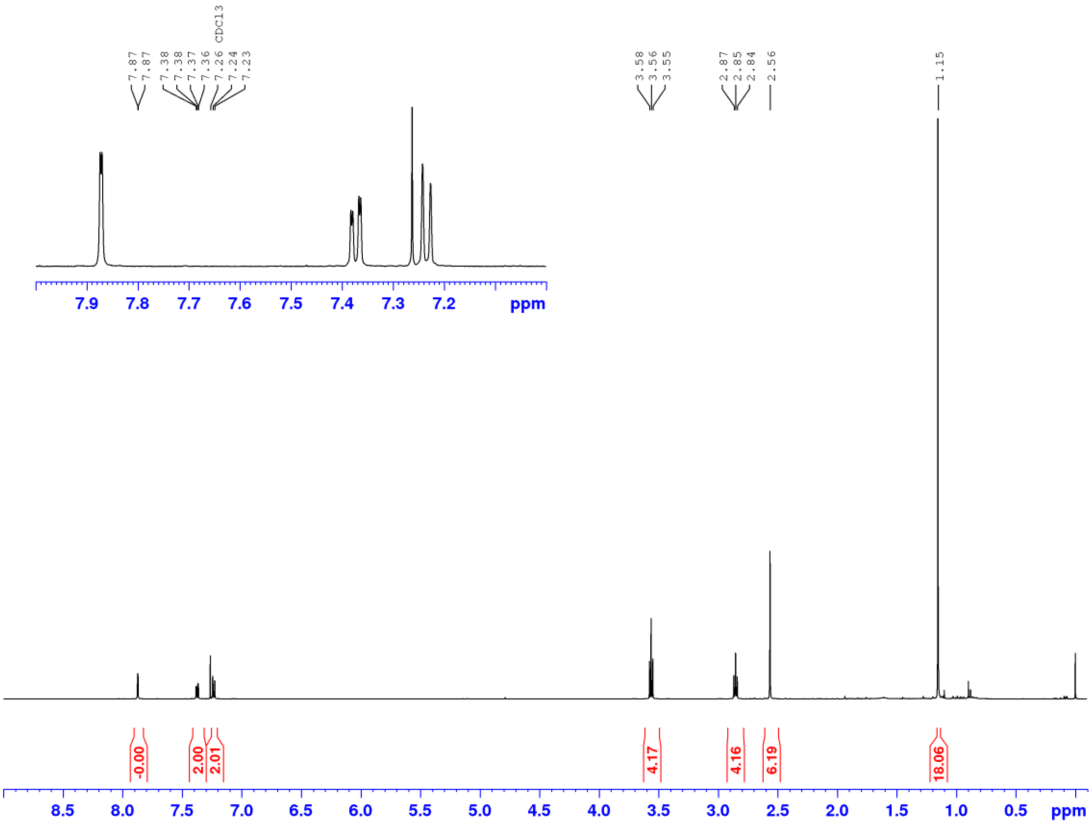


**Figure S3**: $^{1}$H-NMR spectrum of compound **9b** measured in deuterated chloroform.

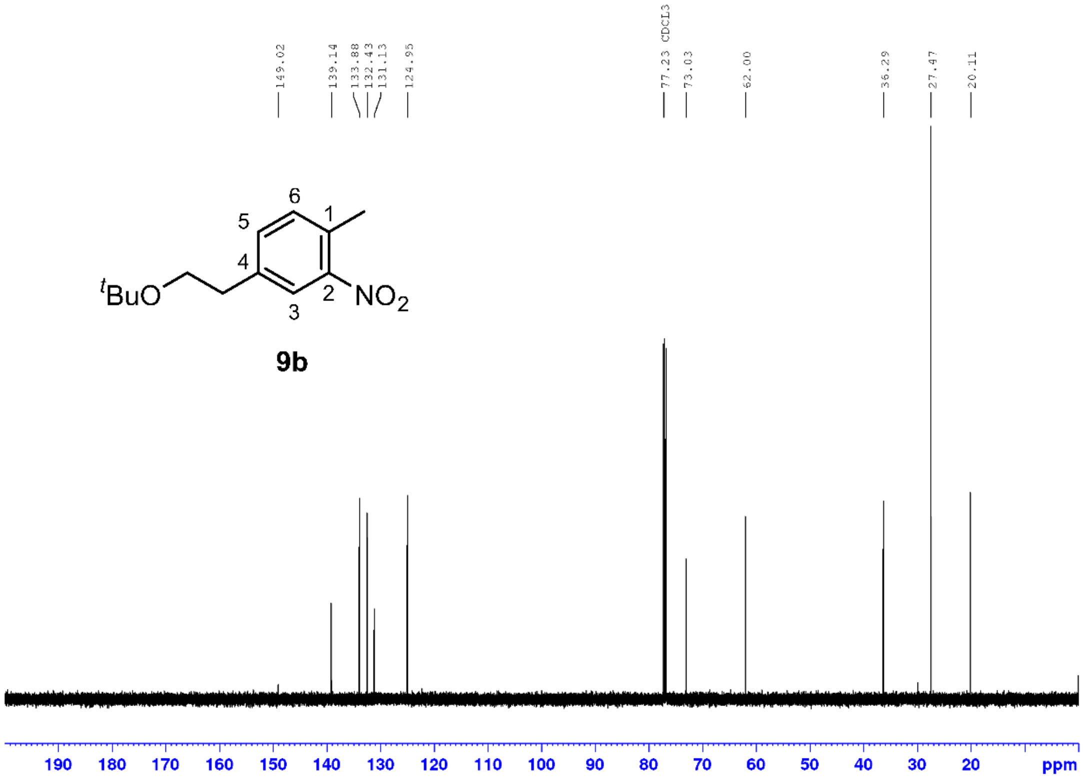


**Figure S4**: $^{13}$C-NMR spectrum of compound **9b** measured in deuterated chloroform.

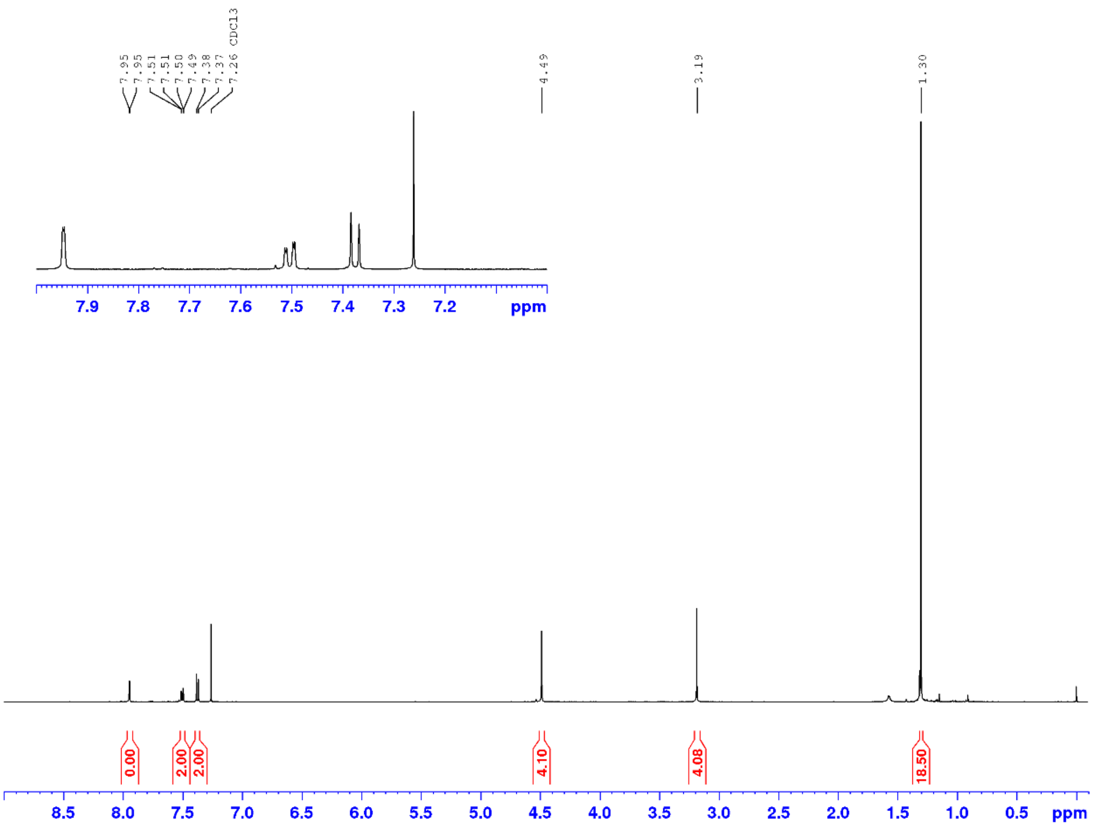

**Figure S5**: $^{1}$H-NMR spectrum of compound **10a** measured in deuterated chloroform.

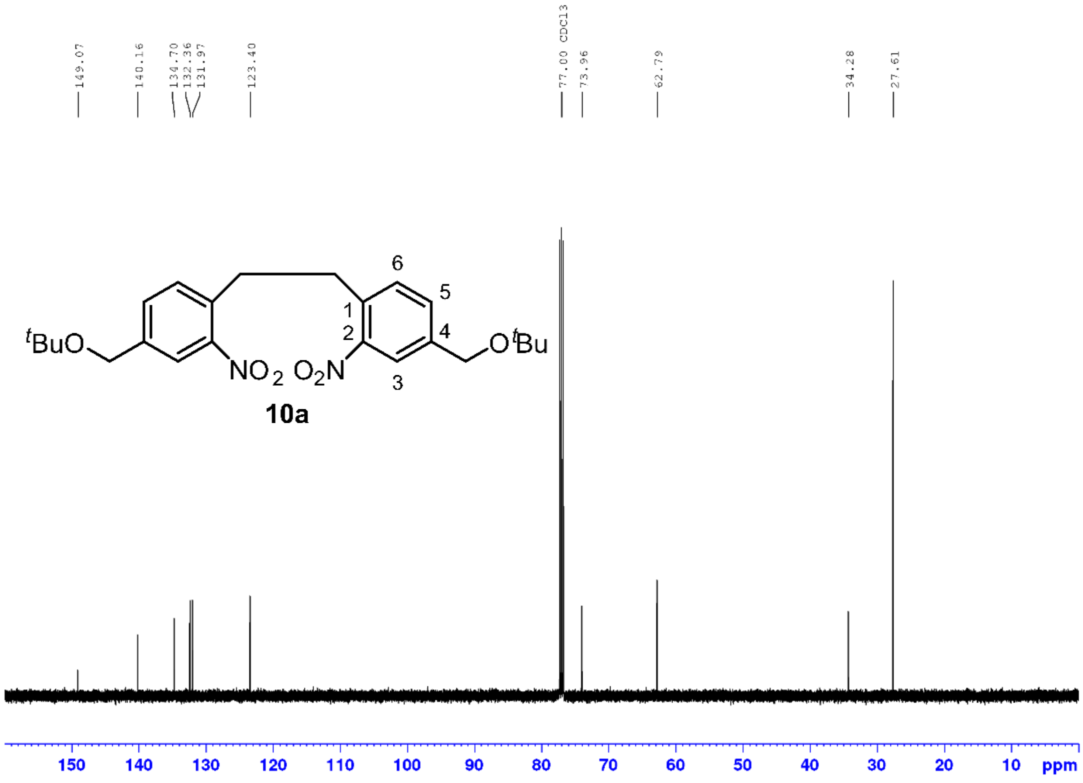

**Figure S6**: $^{13}$C-NMR spectrum of compound **10a** measured in deuterated chloroform.

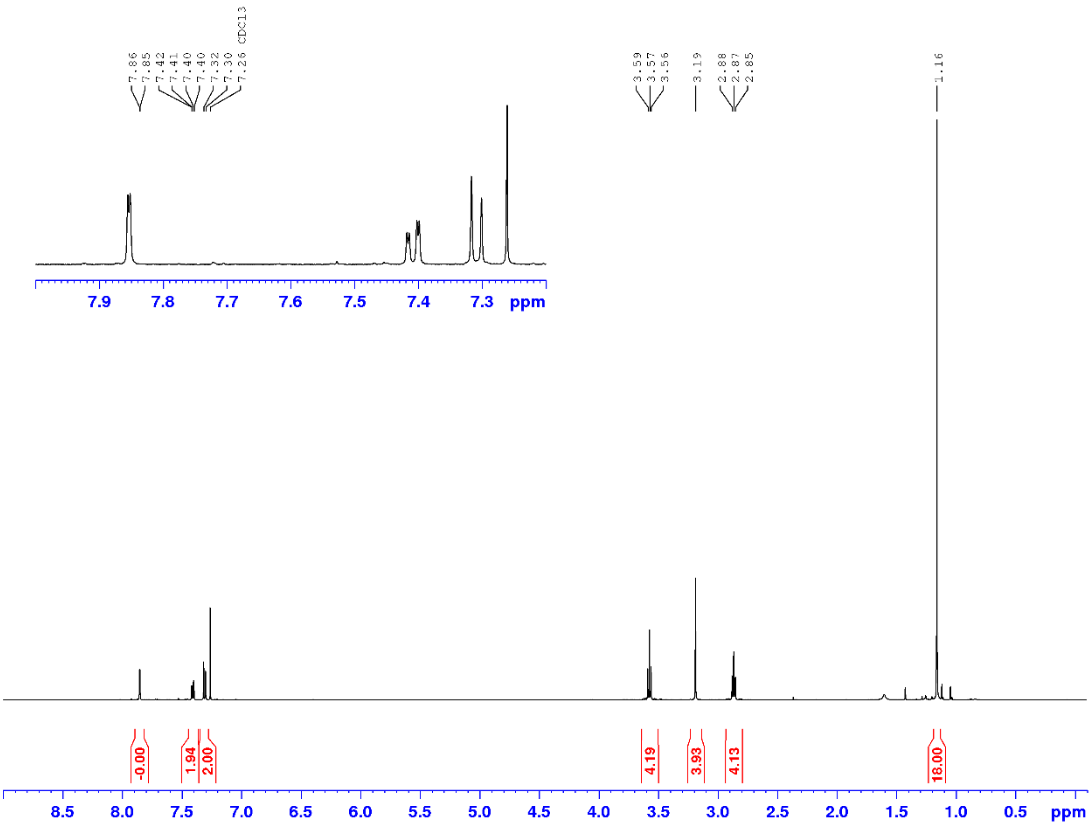


**Figure S7**: $^{1}$H-NMR spectrum of compound **10b** measured in deuterated chloroform.

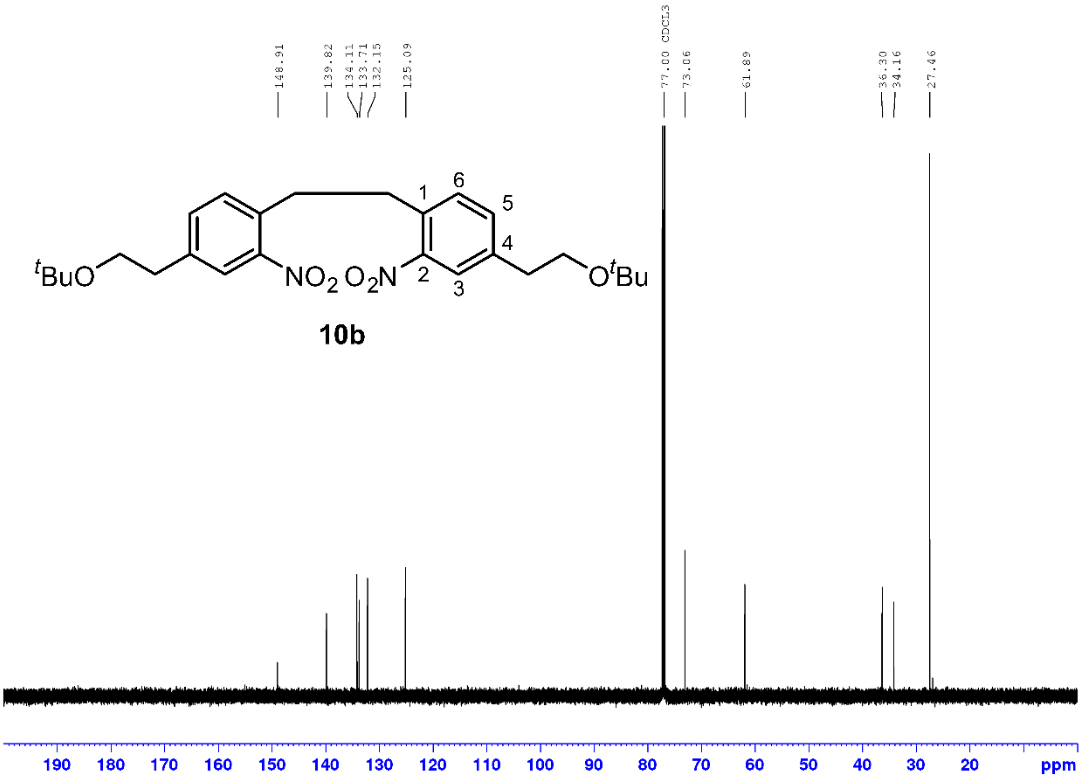


**Figure S8**: $^{13}$C-NMR spectrum of compound **10b** measured in deuterated chloroform.

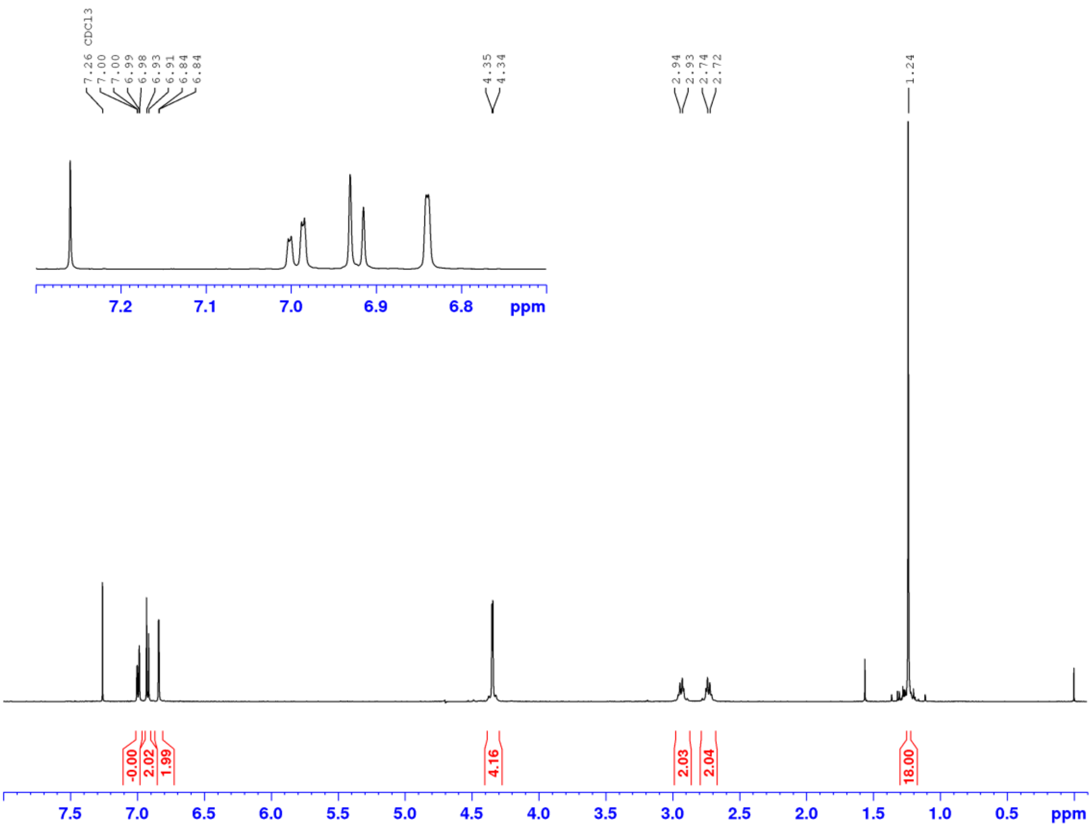


**Figure S9**: $^{1}$H-NMR spectrum of compound **11a** measured in deuterated chloroform.

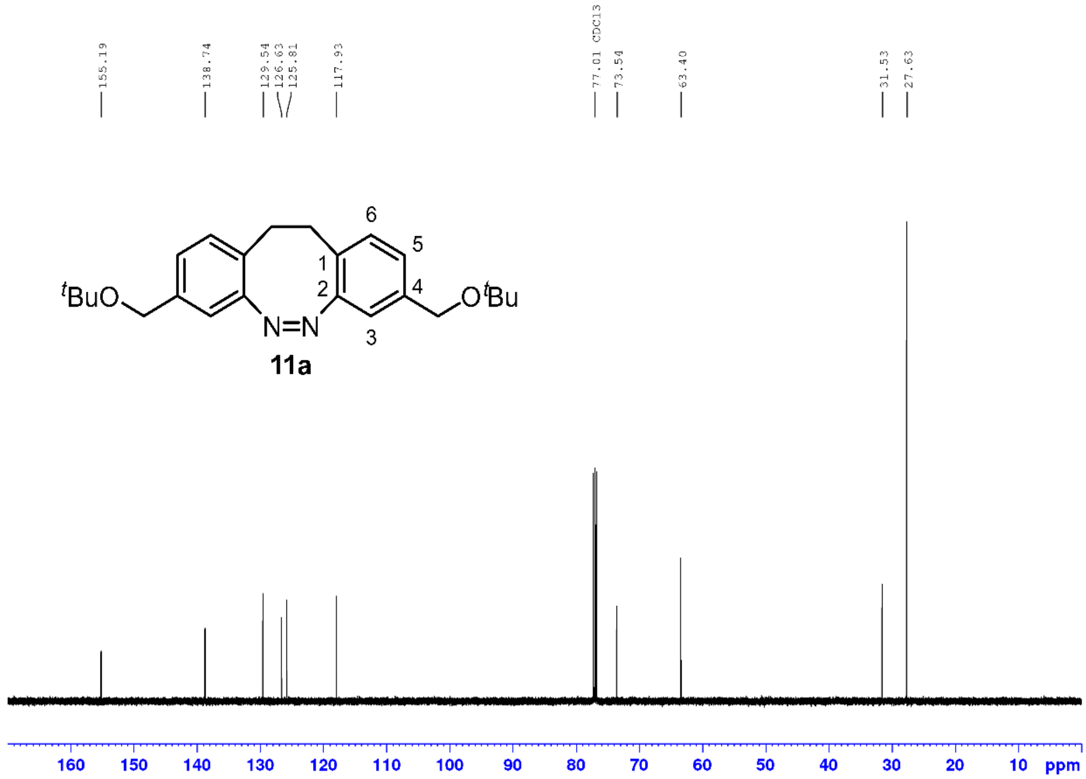


**Figure S10**: $^{13}$C-NMR spectrum of compound **11a** measured in deuterated chloroform.

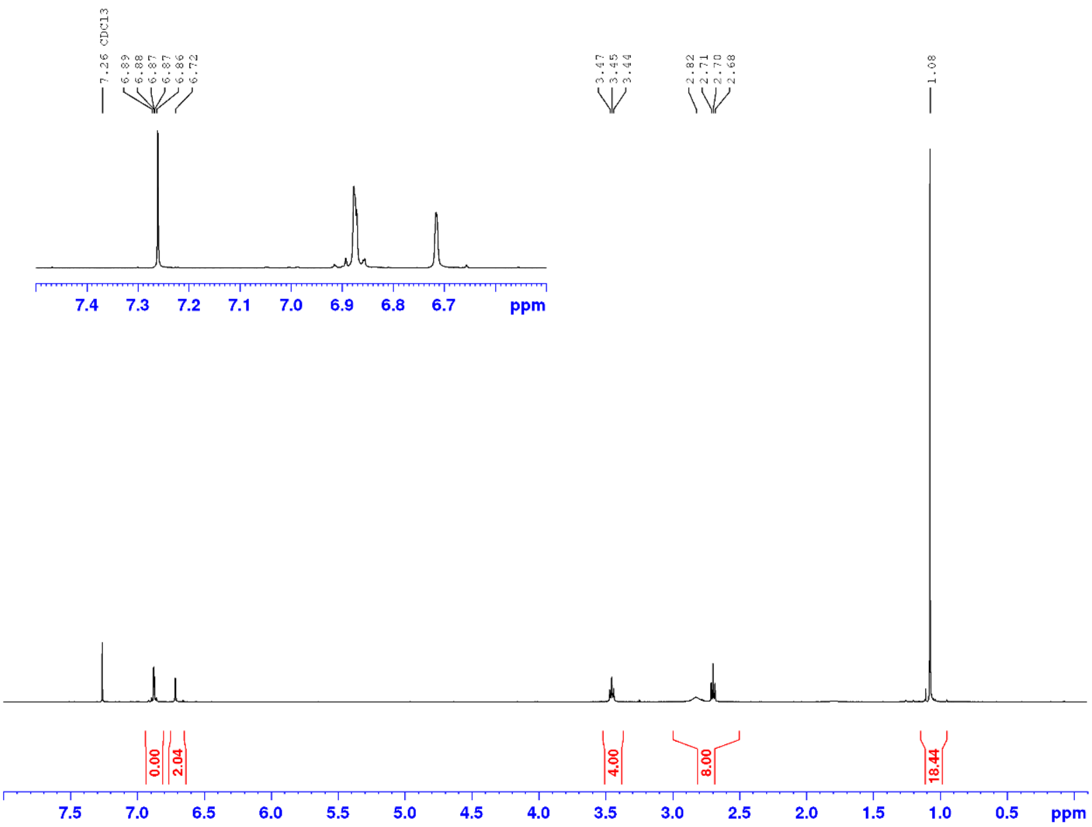


**Figure S11**: $^{1}$H-NMR spectrum of compound **11b** measured in deuterated chloroform.

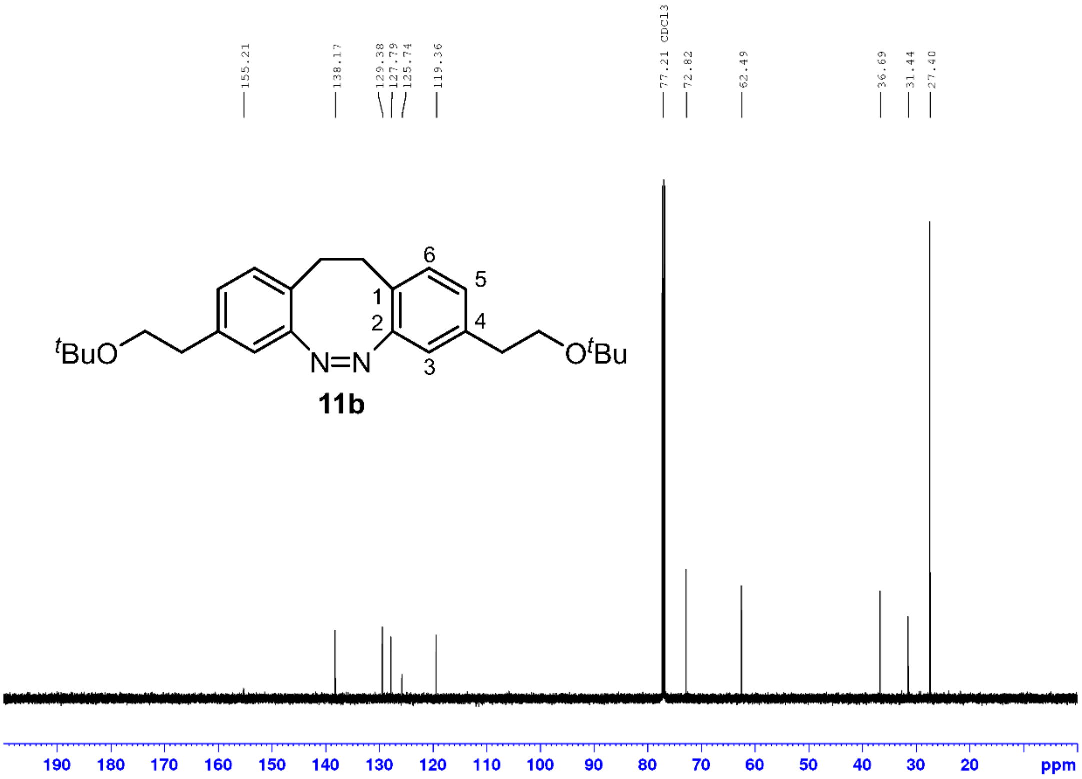


**Figure S12**: $^{13}$C-NMR spectrum of compound **11b** measured in deuterated chloroform.

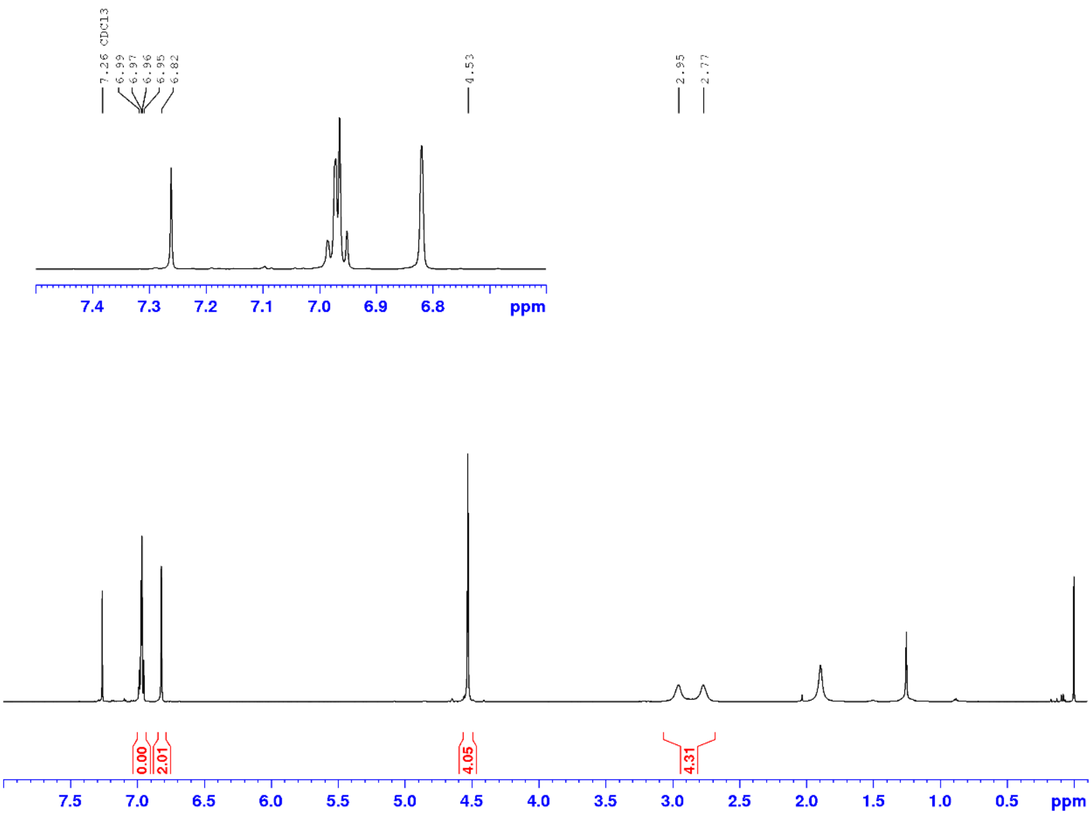


**Figure S13**: $^{1}$H-NMR spectrum of compound **4a** measured in deuterated chloroform.

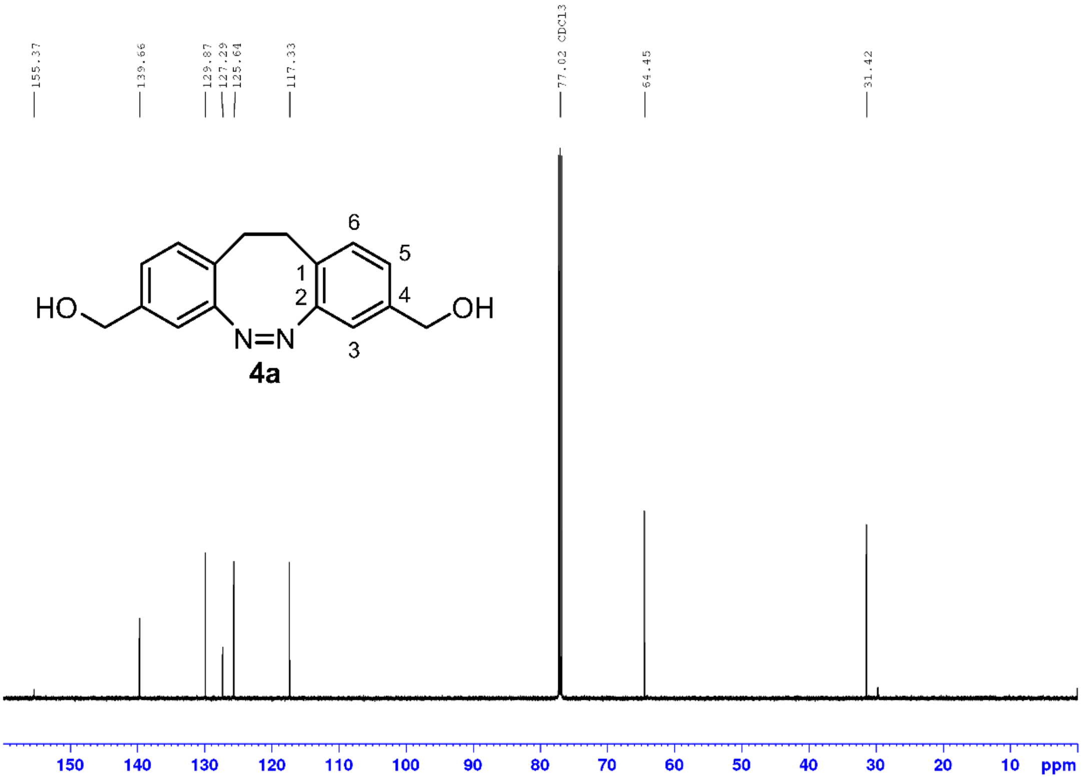


**Figure S14**: $^{13}$C-NMR spectrum of compound **4a** measured in deuterated chloroform.

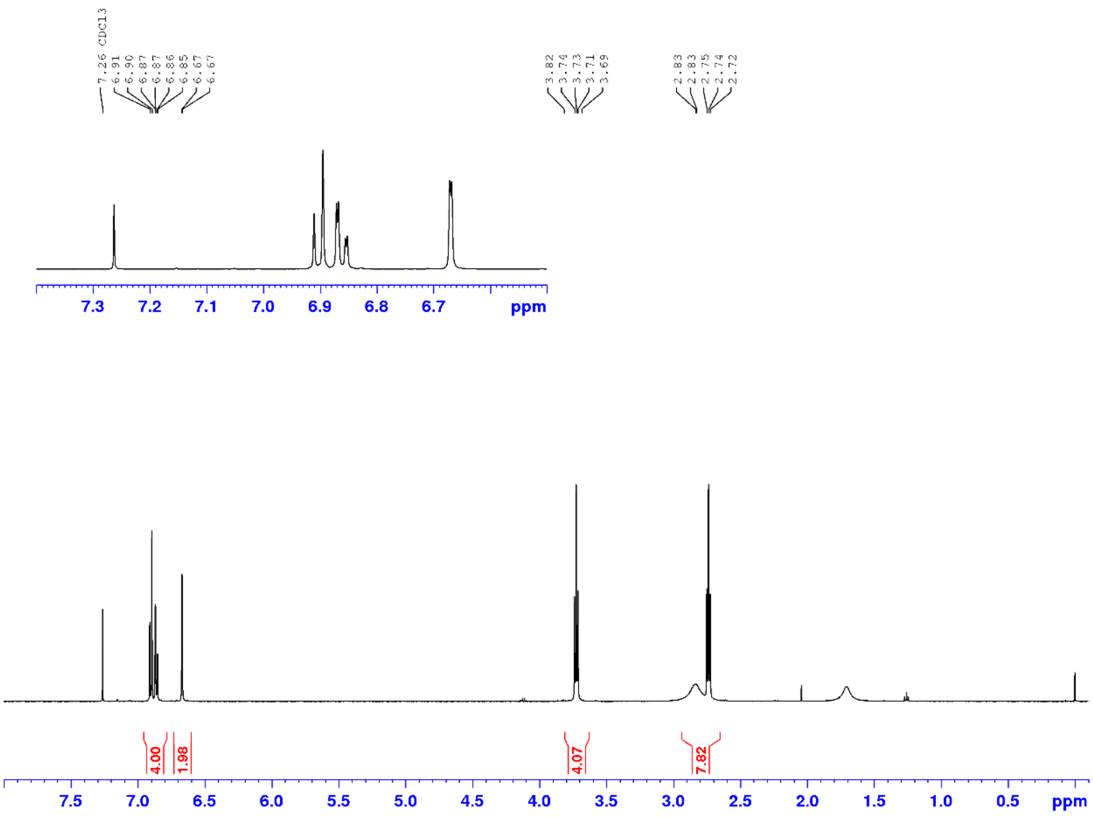

**Figure S15**: $^{1}$H-NMR spectrum of compound **4b** measured in deuterated chloroform.

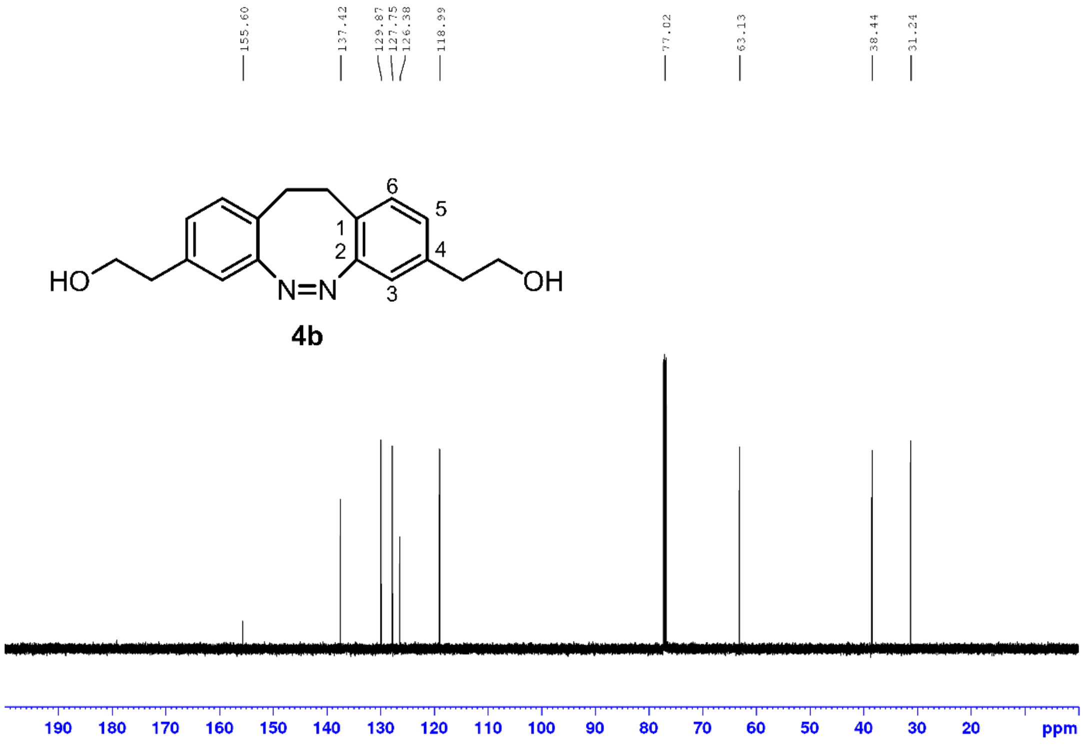


**Figure S16**: $^{13}$C-NMR spectrum of compound **4b** measured in deuterated chloroform.

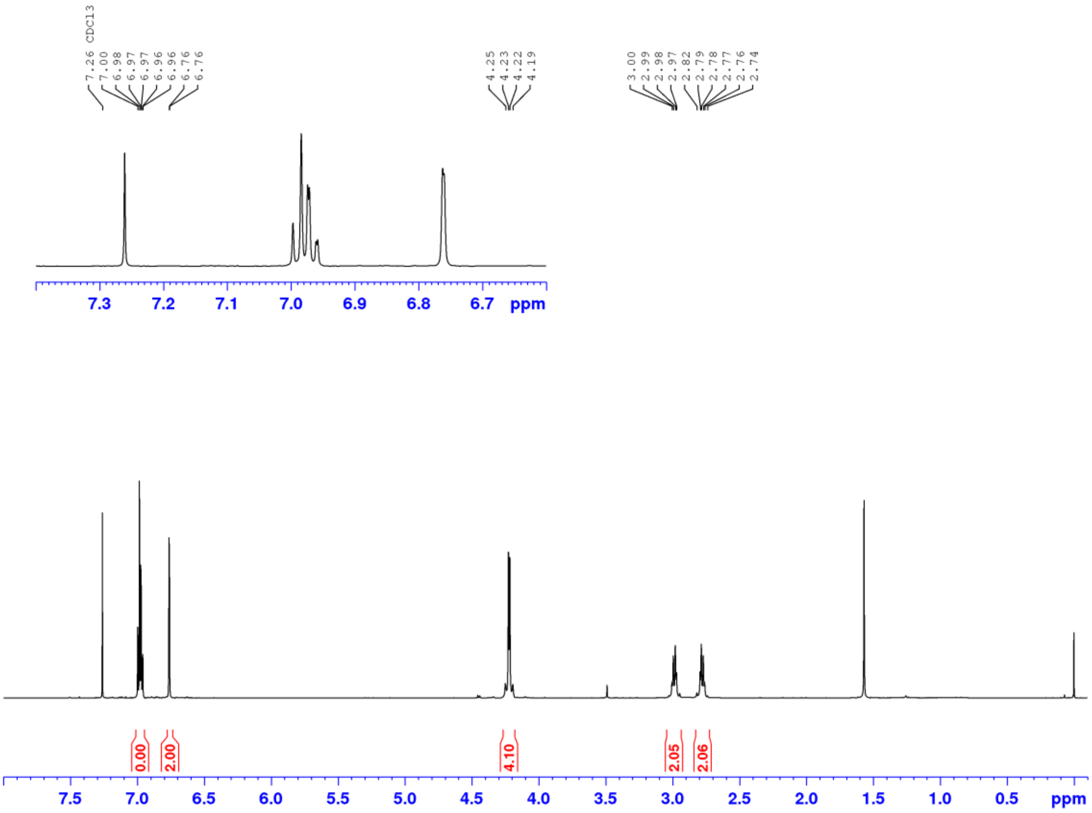


**Figure S17**: $^{1}$H-NMR spectrum of compound **5a** measured in deuterated chloroform.

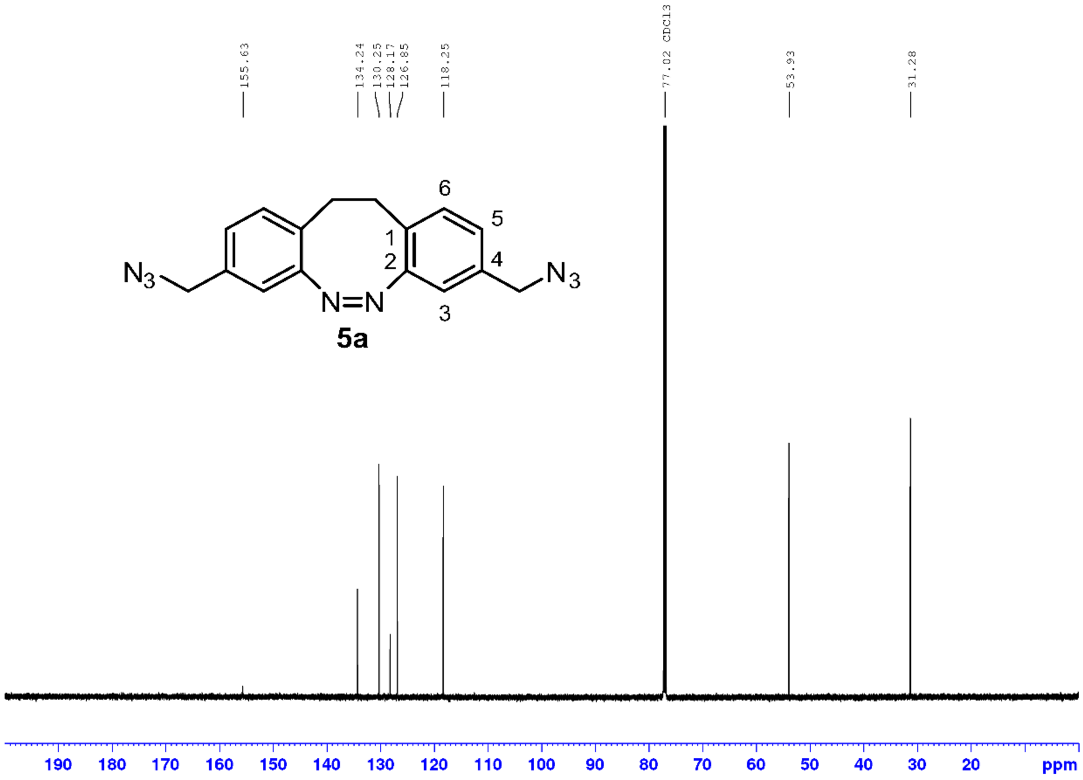


**Figure S18**: $^{13}$C-NMR spectrum of compound **5a** measured in deuterated chloroform.

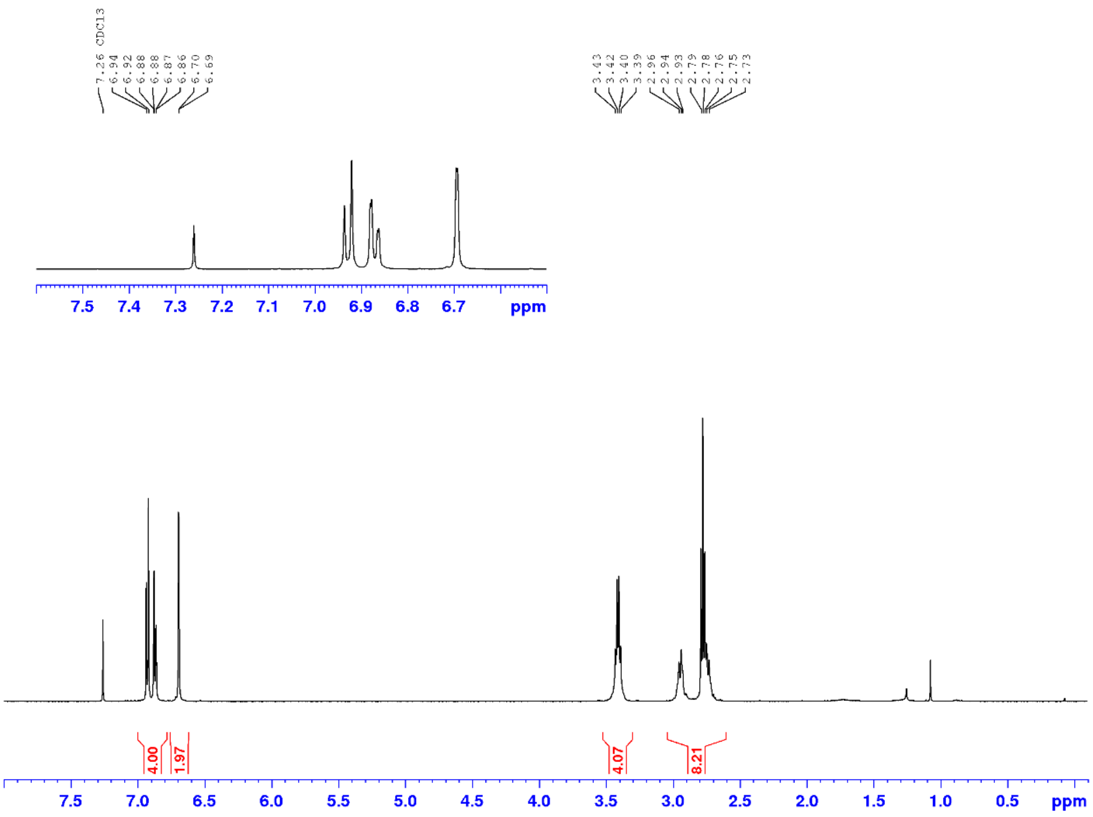

**Figure S19**: $^{1}$H-NMR spectrum of compound **5b** measured in deuterated chloroform.

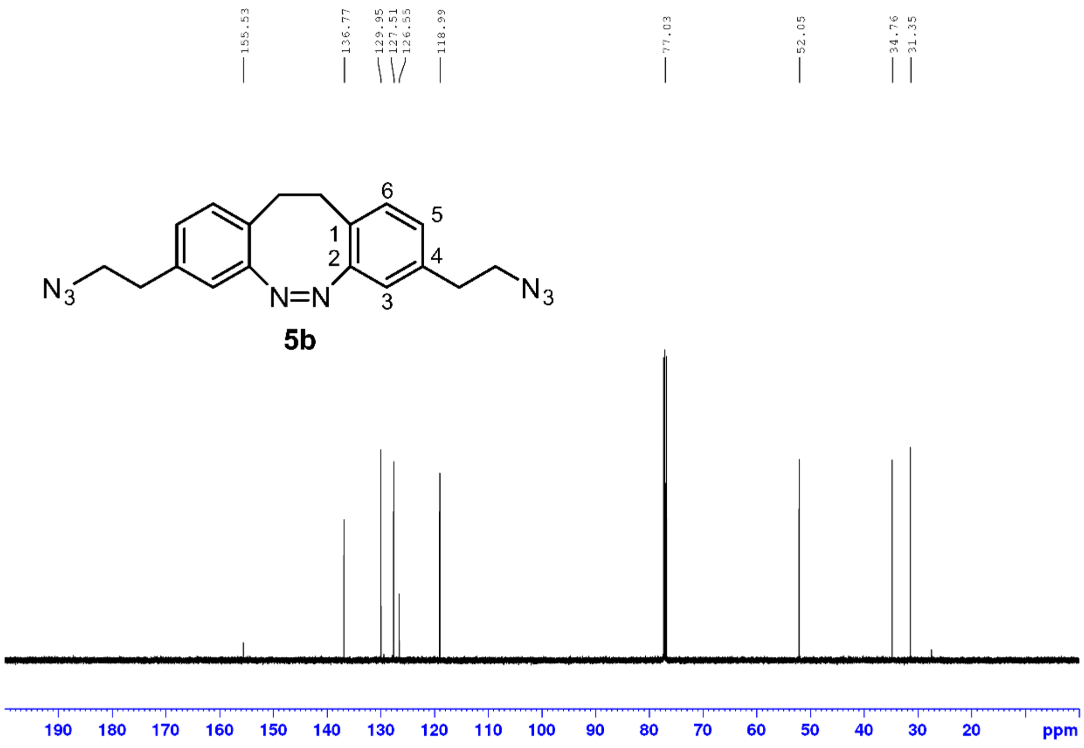

**Figure S20**: $^{13}$C-NMR spectrum of compound **5b** measured in deuterated chloroform.

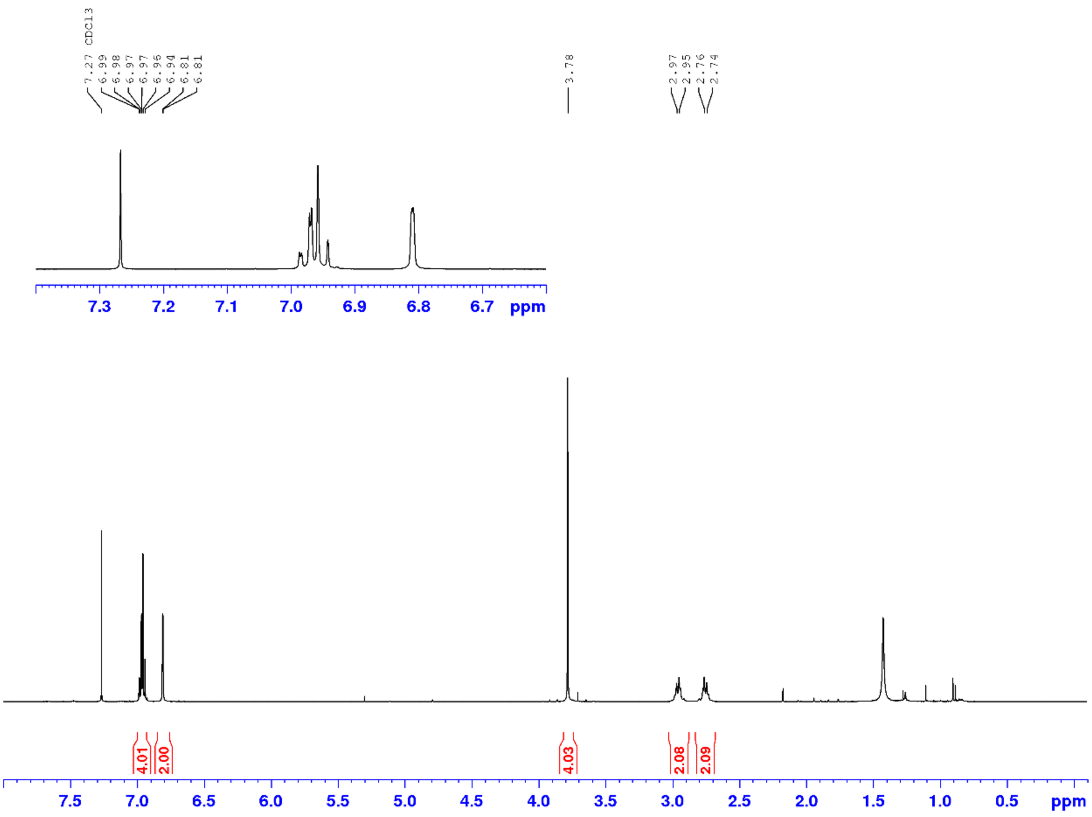

**Figure S21**: $^{1}$H-NMR spectrum of compound **6a** measured in deuterated chloroform.

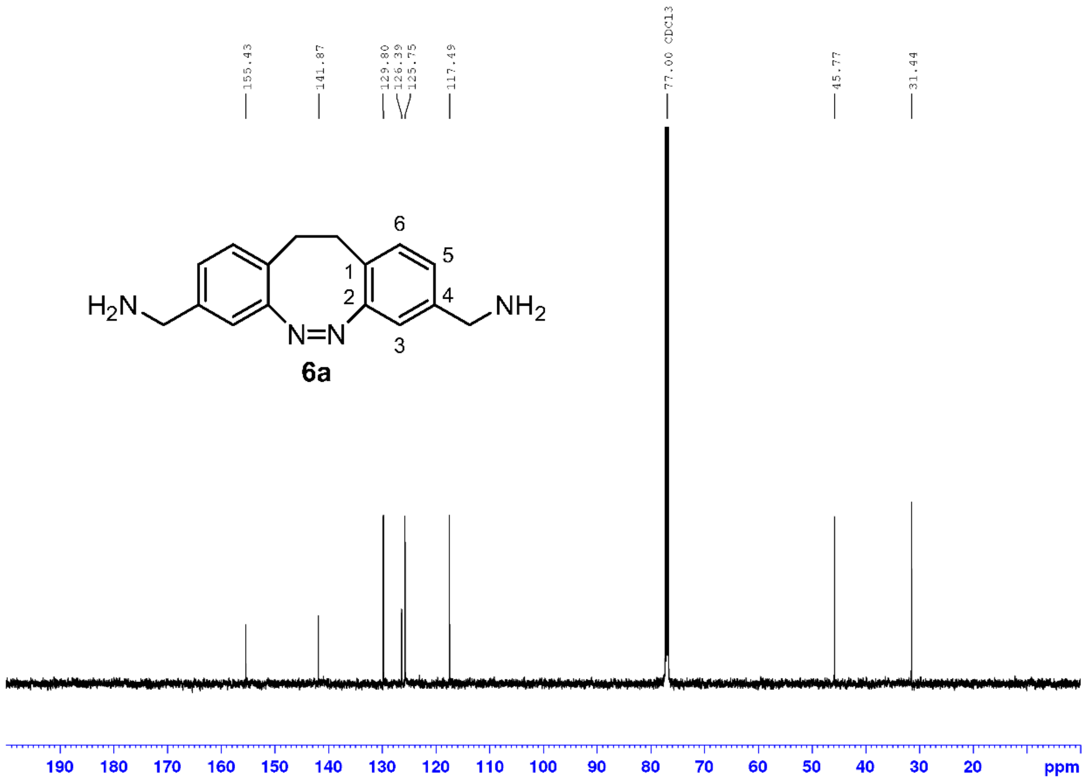


**Figure S22**: $^{13}$C-NMR spectrum of compound **6a** measured in deuterated chloroform.

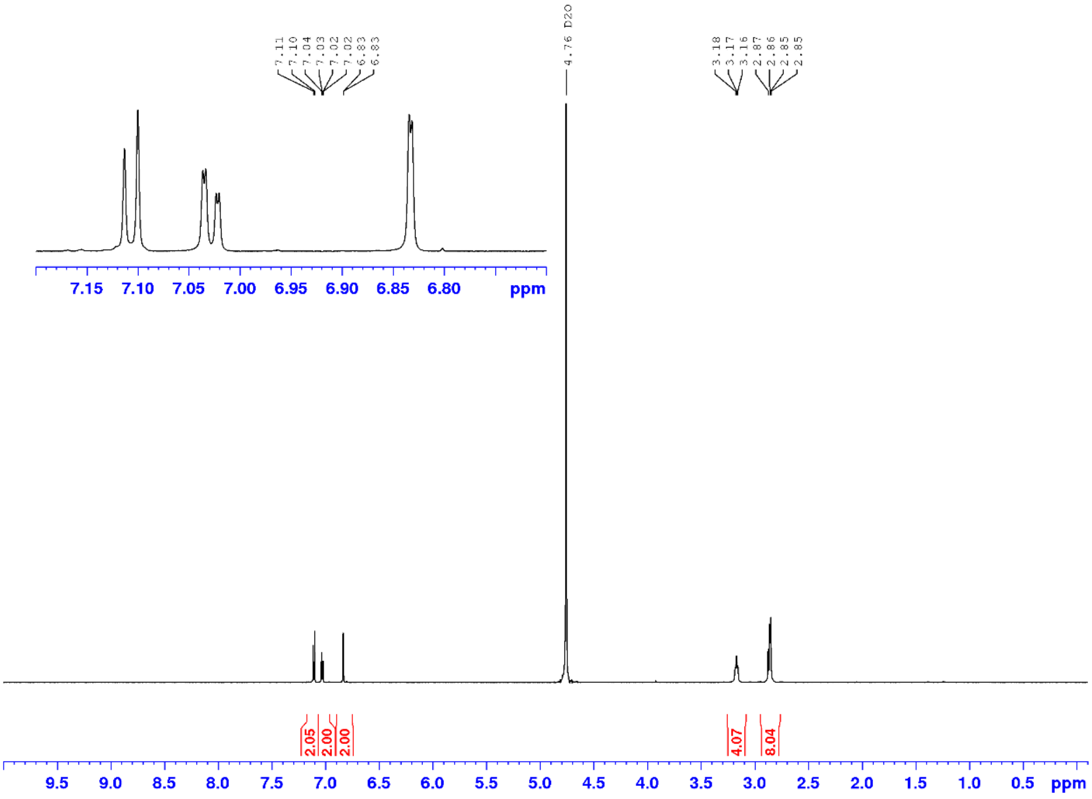


**Figure S23**: $^{1}$H-NMR spectrum of compound **6b** measured in deuterated water.

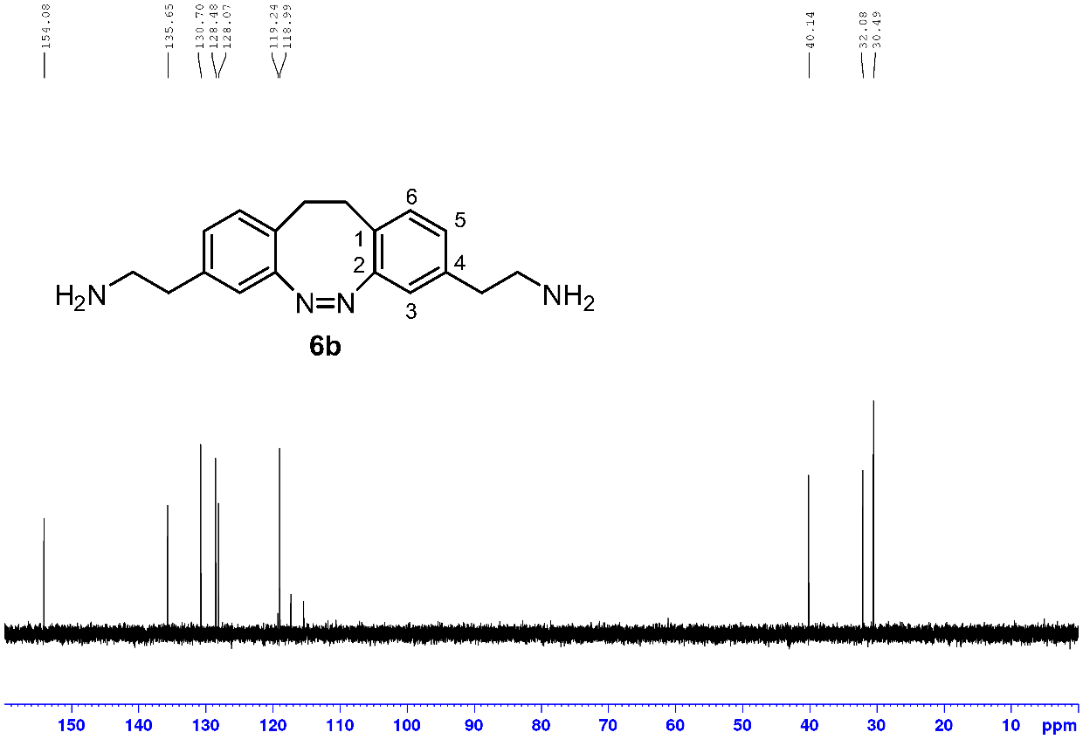


**Figure S24**: $^{13}$C-NMR spectrum of compound **6b** measured in deuterated water.

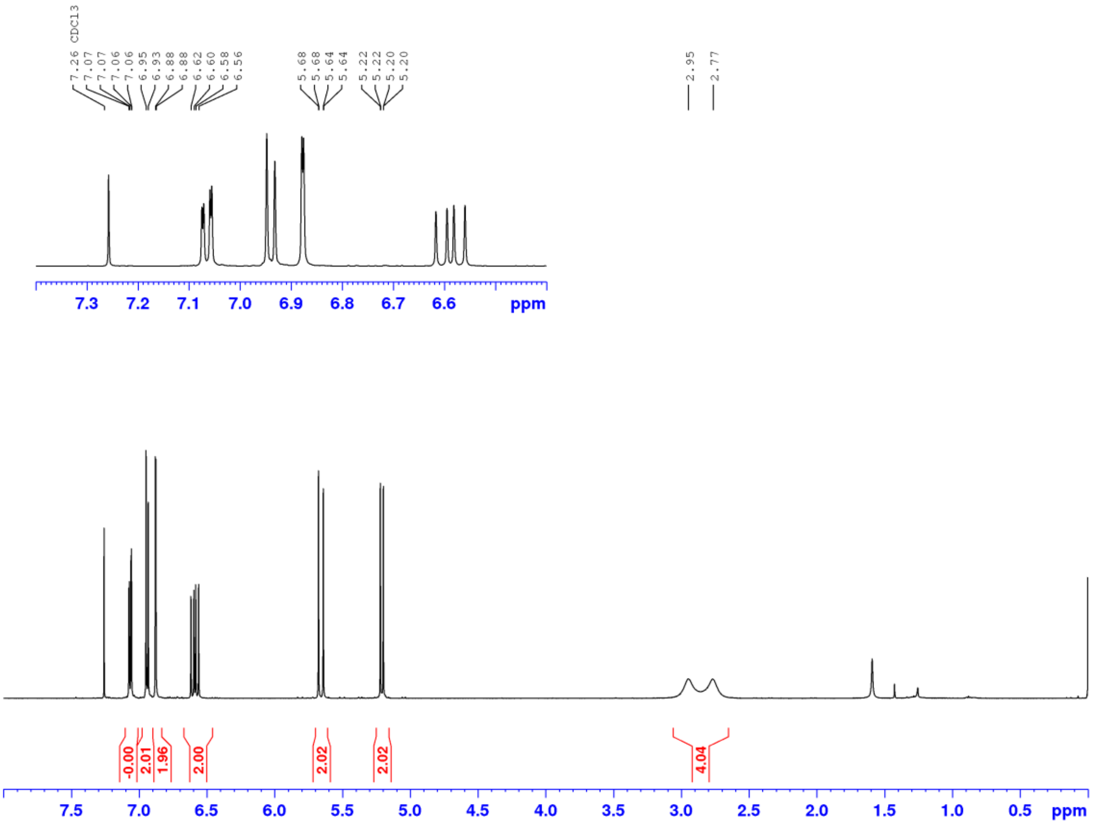


**Figure S25**: $^{1}$H-NMR spectrum of compound **7** measured in deuterated chloroform.

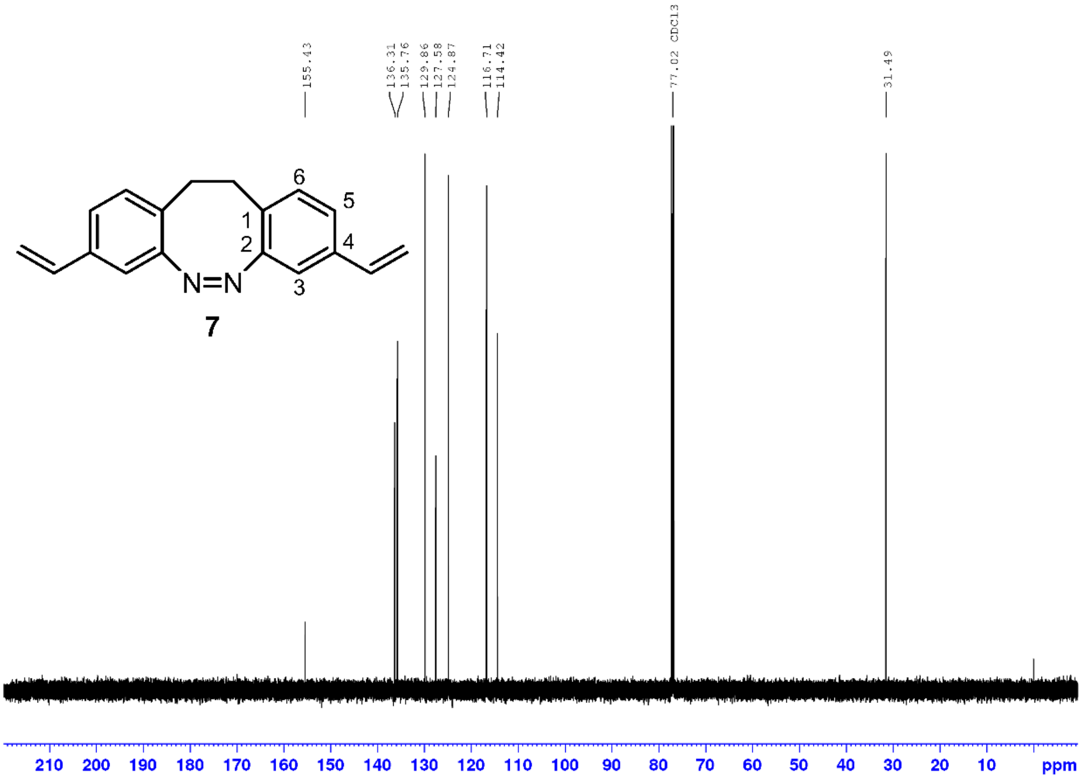


**Figure S26**: $^{13}$C-NMR spectrum of compound **7** measured in deuterated chloroform.

## IV. UV–vis spectra

The functionalized diazocines **1**–**7**,**11a** and **11b** were dissolved in acetonitrile (0.05 mM) under exclusion of light and UV–vis *cis* spectra (black) were recorded. Then the solutions were irradiated at 385 nm for 2 min and the UV–vis PSS spectra (red) were measured.

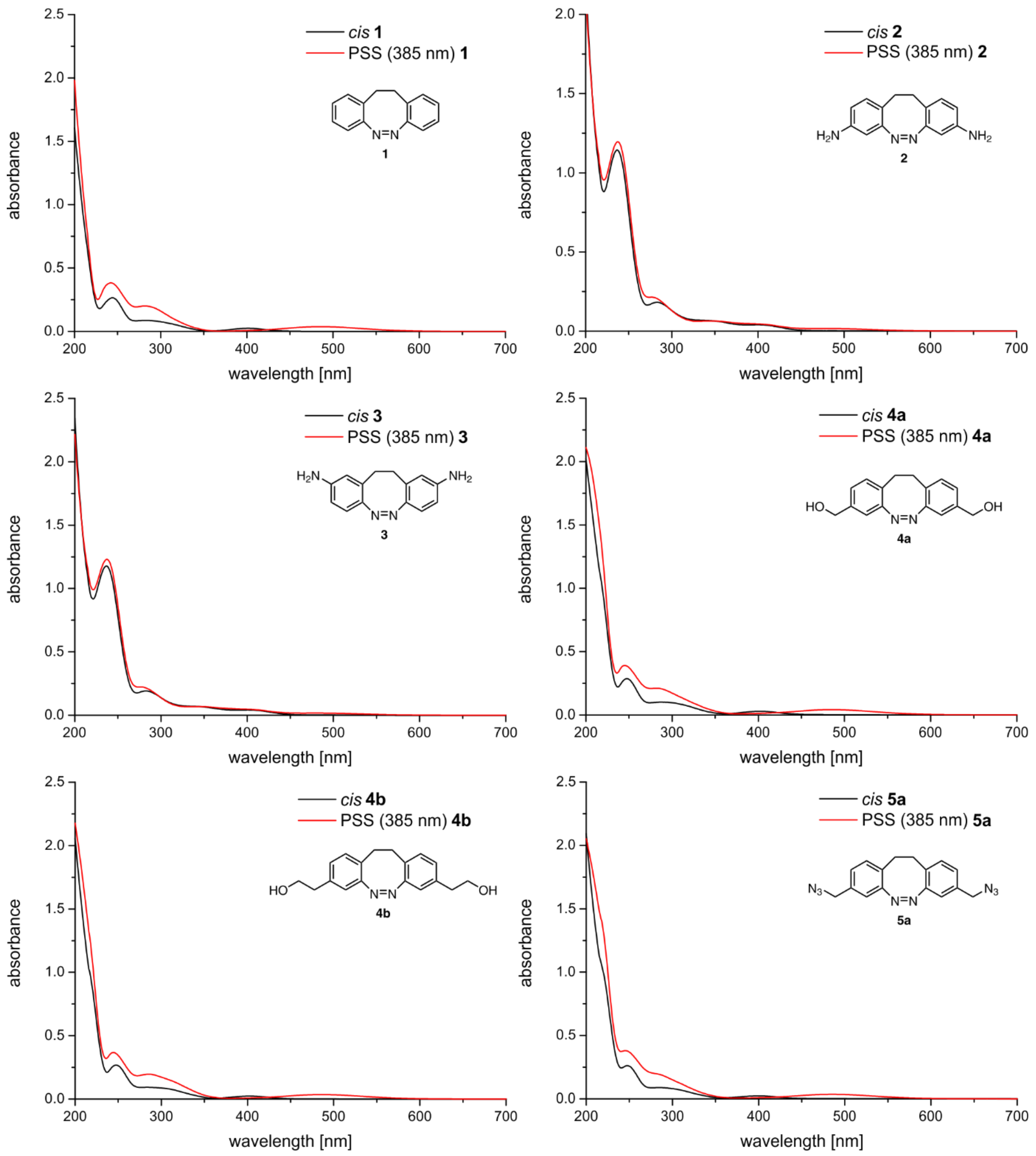


**Figure S27**: UV–vis spectra of functionalized diazocines **1**, **2**, **3**, **4a**, **4b** and **5a** spectra measured at 25 °C in acetonitrile (0.05 mM). The black line is the spectrum of the pure *cis* compound and in red the PSS after irradiation at 385 nm.

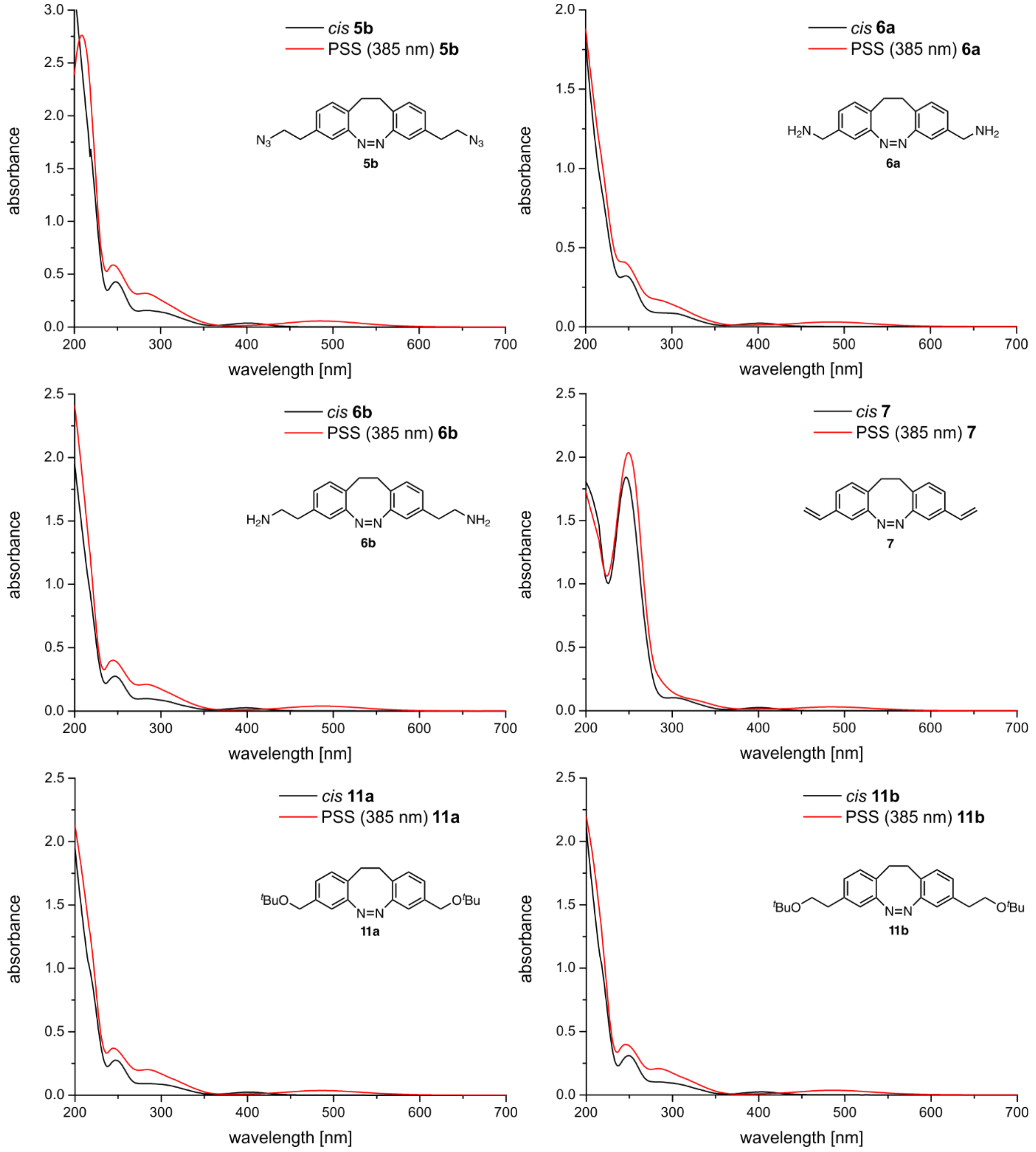


**Figure S28**: UV–vis of functionalized diazocines **5b**, **6a**, **6b**, **7**, **11a** and **11b** spectra measured at 25 °C in acetonitrile 0.05mM. The black line is the spectrum of the pure *cis* compound and in red the PSS after irradiation at 385 nm.

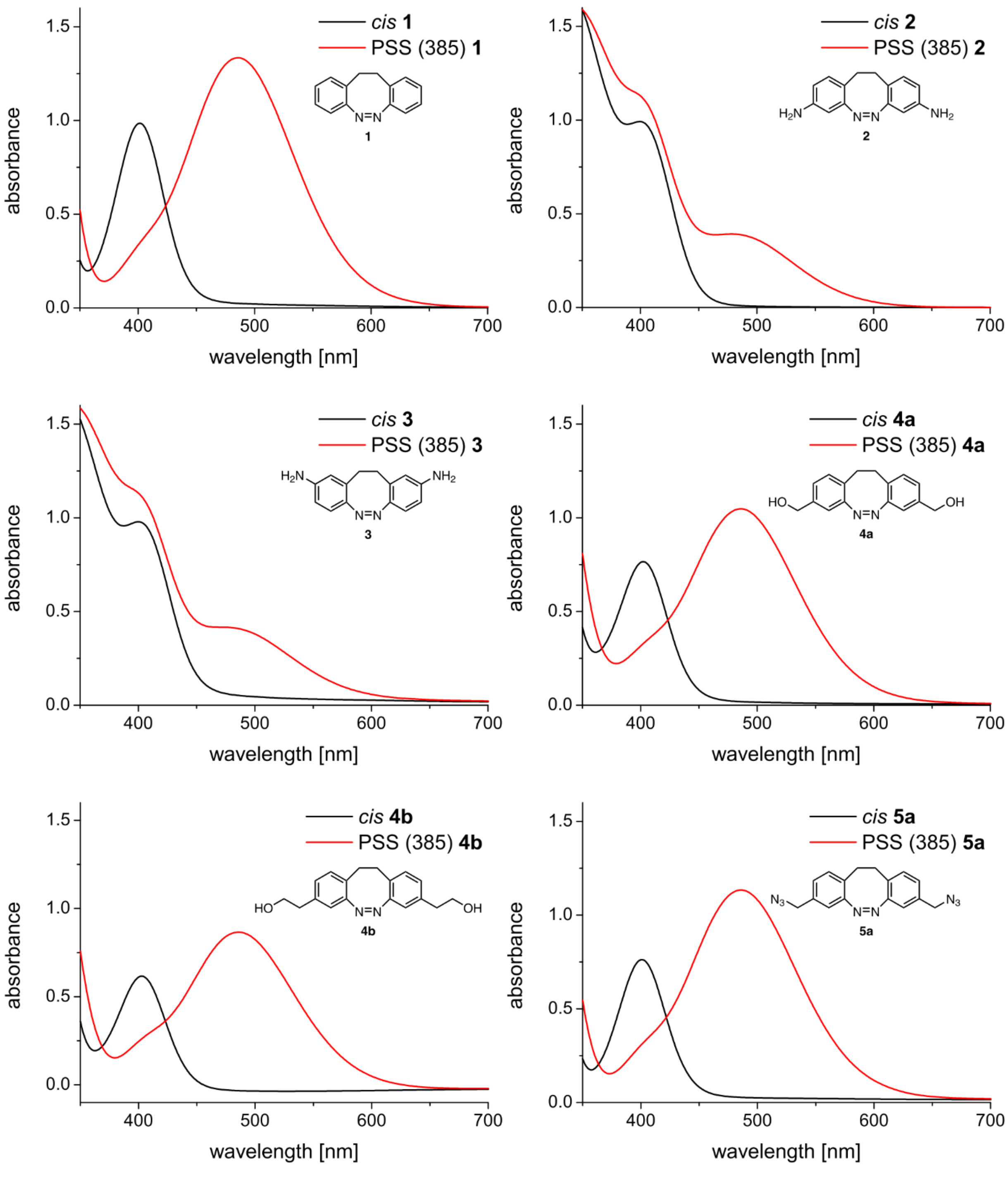


**Figure S29**: UV–vis spectra of functionalized diazocines **1** (2.0 mM), **2** (1.3 mM), **3** (1.3 mM), **4a** (1.5 mM), **4b** (1.5 mM) and **5a** (1.0 mM) spectra measured at 25 °C in acetonitrile. The black line is the spectrum of the pure *cis* compound and the red the PSS after irradiation at 385 nm.

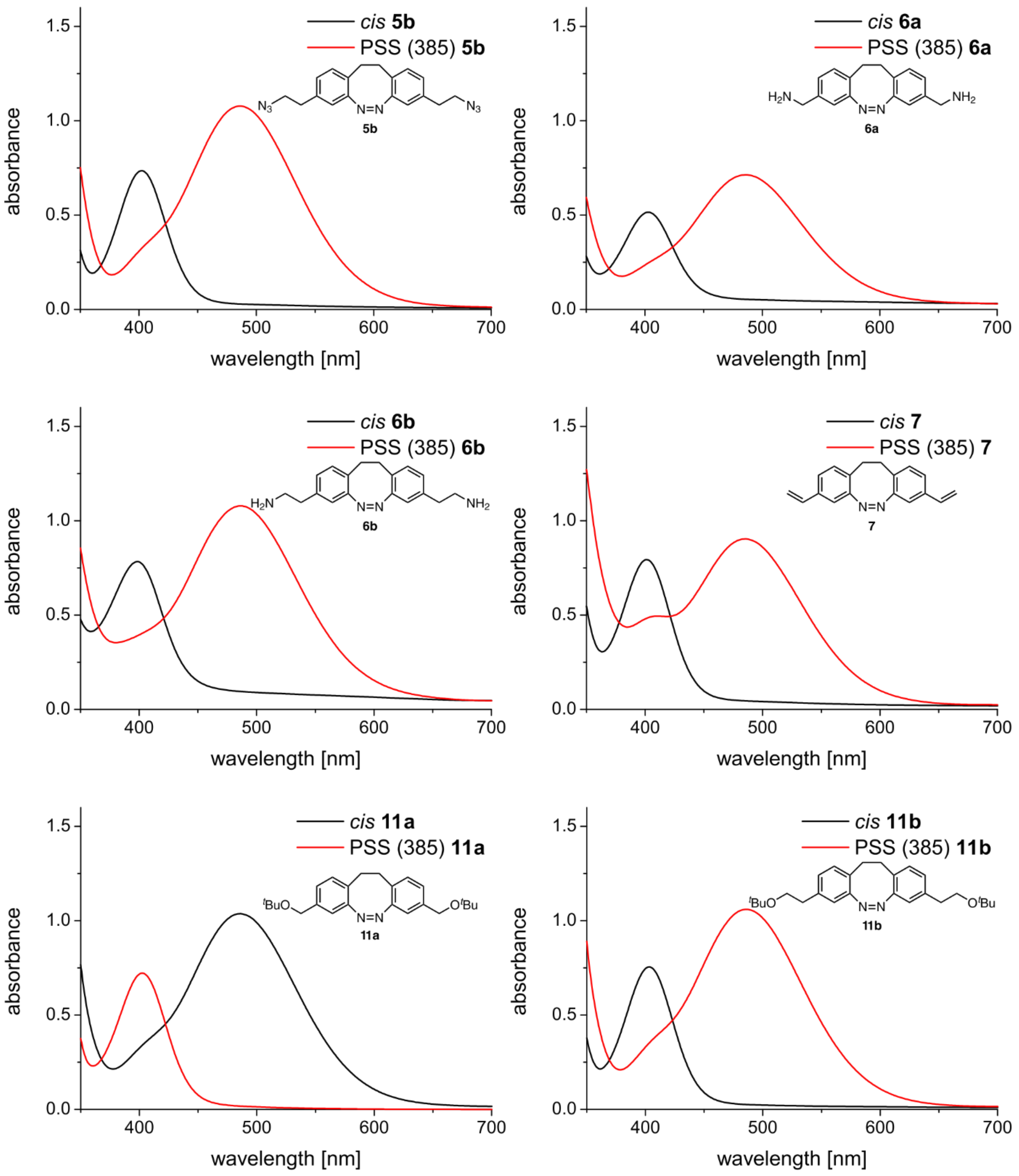


**Figure S30**: UV–vis of functionalized diazocines **5b** (1.7 mM), **6a** (1.2 mM), **6b** (1.6 mM), **7** (1.6 mM), **11a** (1.5 mM) and **11b** (1.5 mM) spectra measured at 25 °C in acetonitrile. The black line is the spectrum of the pure *cis* compound and in red the PSS after irradiation at 385 nm.

## V. Photochemical experiments

### Photostationary states (PSS 385 nm)

Photostationary states (PSS) were investigated by $^{1}$H-NMR spectroscopy and measured in acetonitrile-$d_3$ at 300 K. Samples of 10 mM were prepared under exclusion of light and measured without irradiation. Afterwards the samples were irradiated for 5 min at 385 nm outside the NMR spectrometer and under ventilation. After recording the samples were irradiated again under the same conditions to guarantee no further isomerization and recorded a second time. Integration of the relevant signals in the $^{1}$H-NMR spectra leads to the PSS at 385 nm.

### NMR spectra at PSS (385 nm)

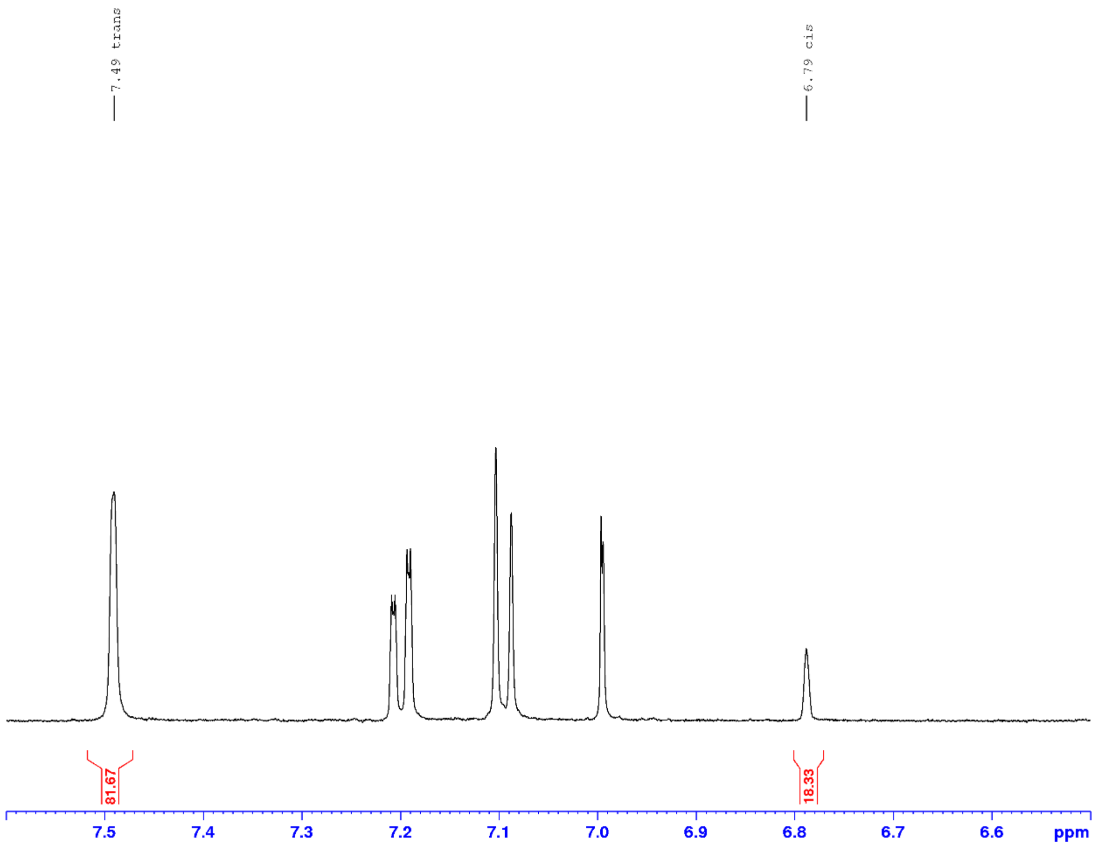


**Figure S31**: $^{1}$H-NMR spectrum of compound **11a** at PSS (385 nm).

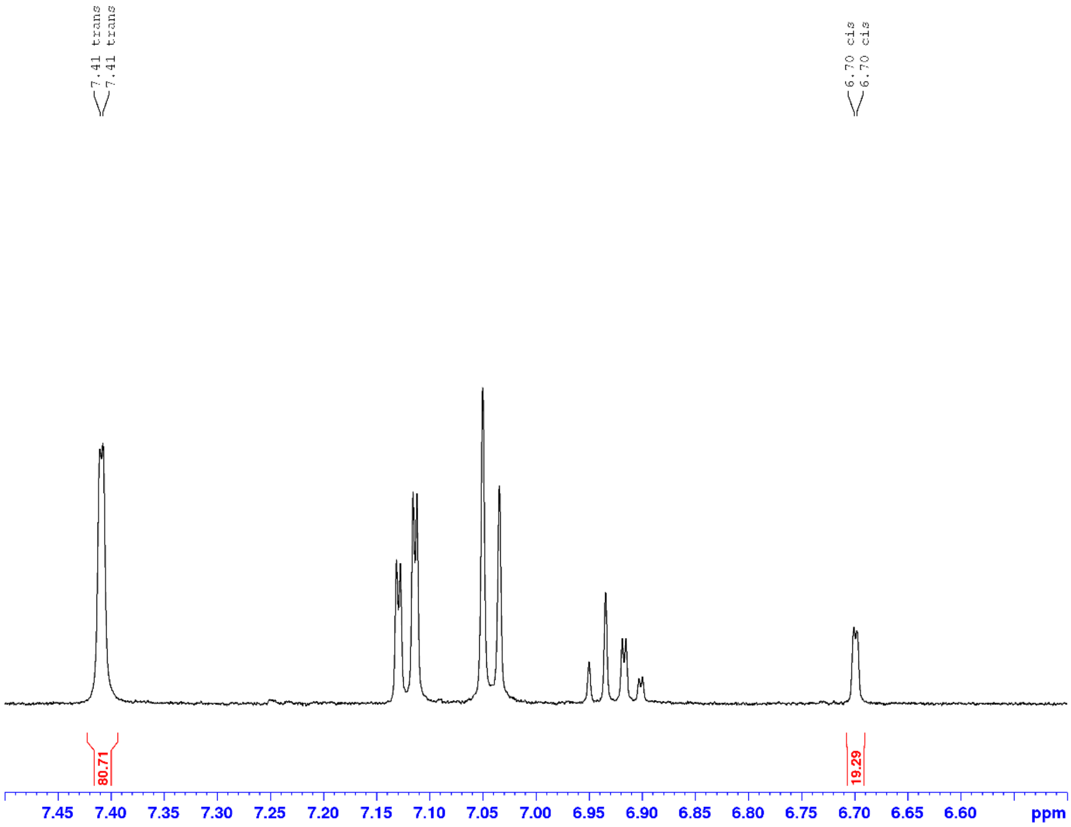


**Figure S32**: $^{1}$H-NMR spectrum of compound **11b** at PSS (385 nm).

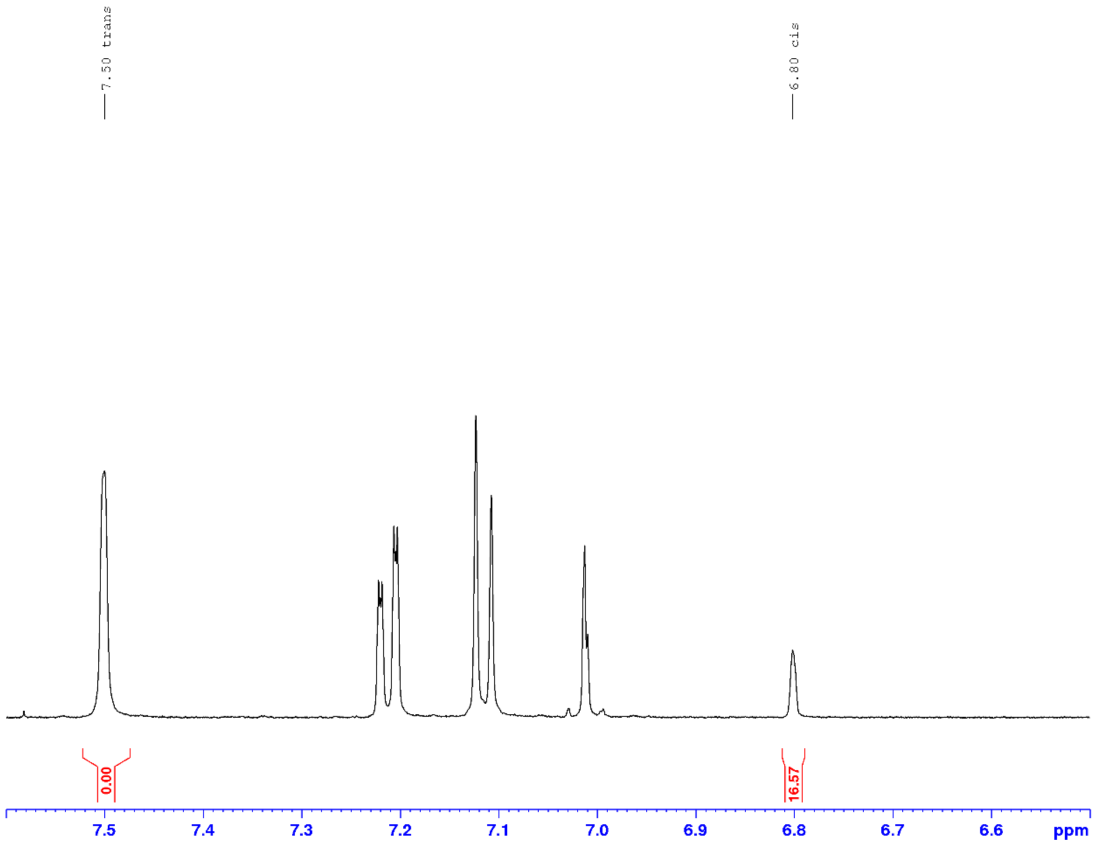


**Figure S33**: $^{1}$H-NMR spectrum of compound **4a** at PSS (385 nm).

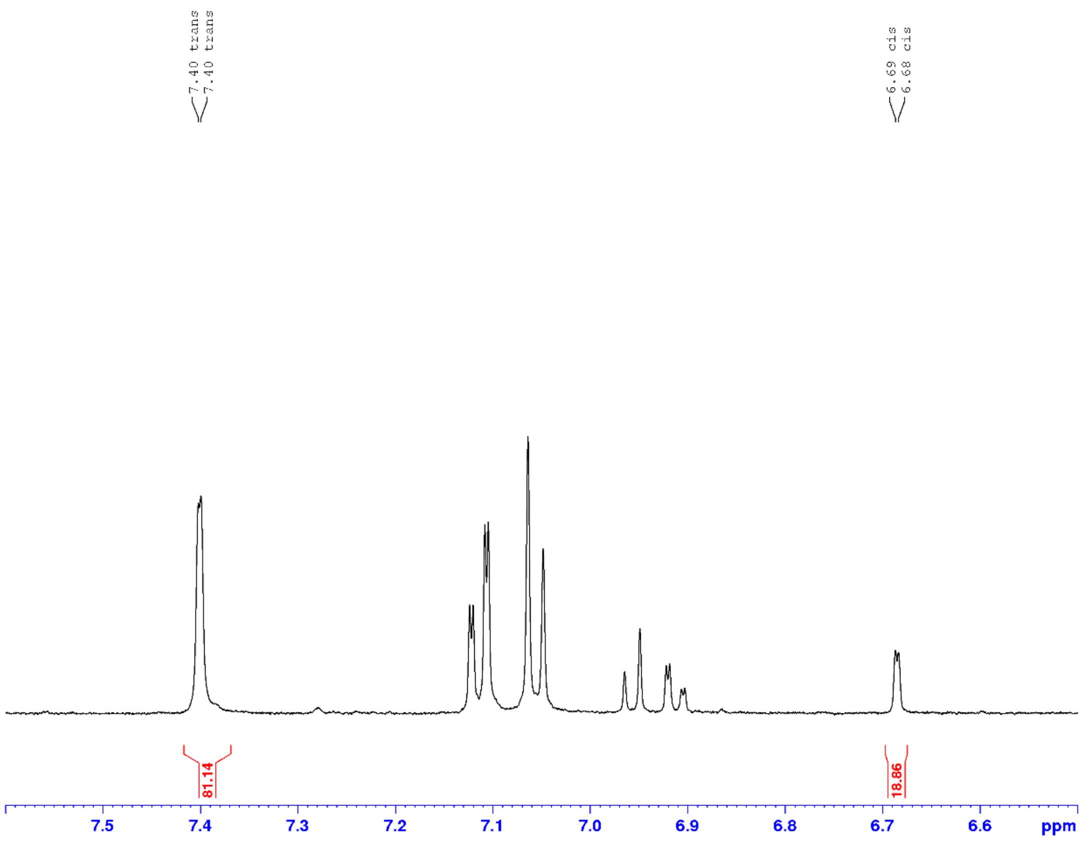


**Figure S34**: $^{1}$H-NMR spectrum of compound **4b** at PSS (385 nm).

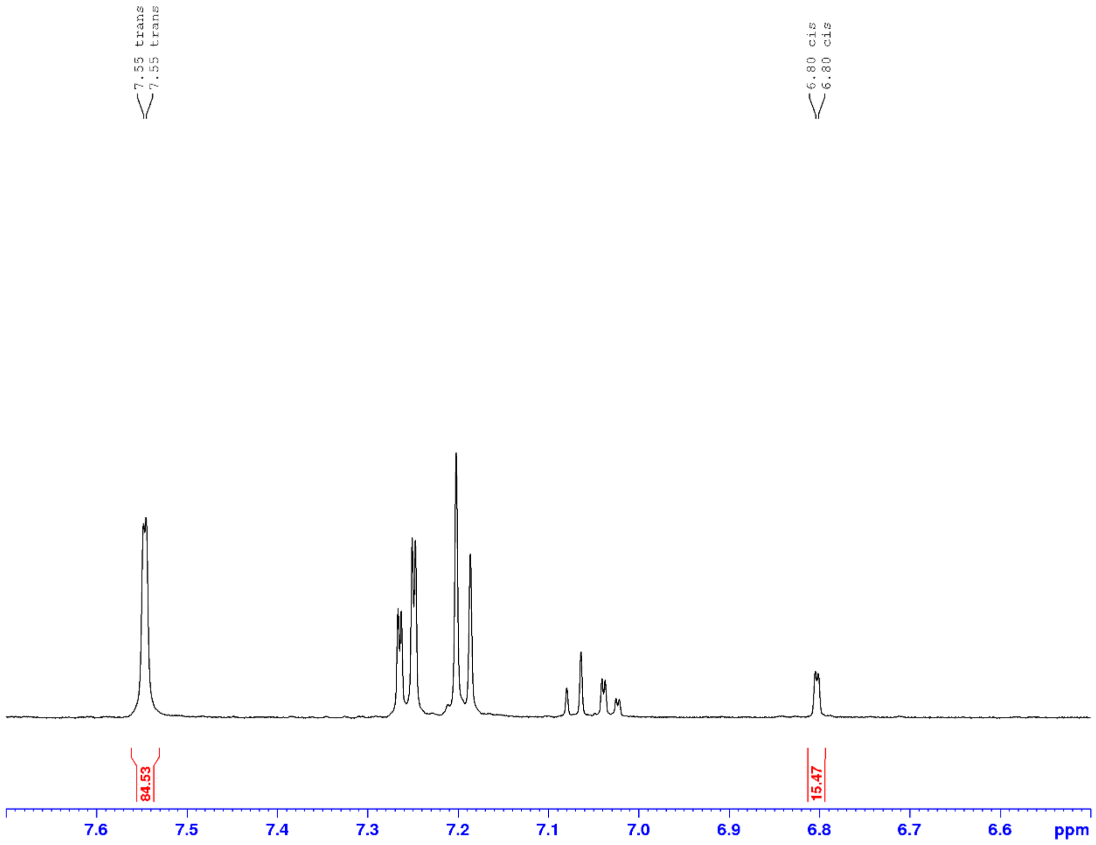


**Figure S35**: $^{1}$H-NMR spectrum of compound **5a** at PSS (385 nm).

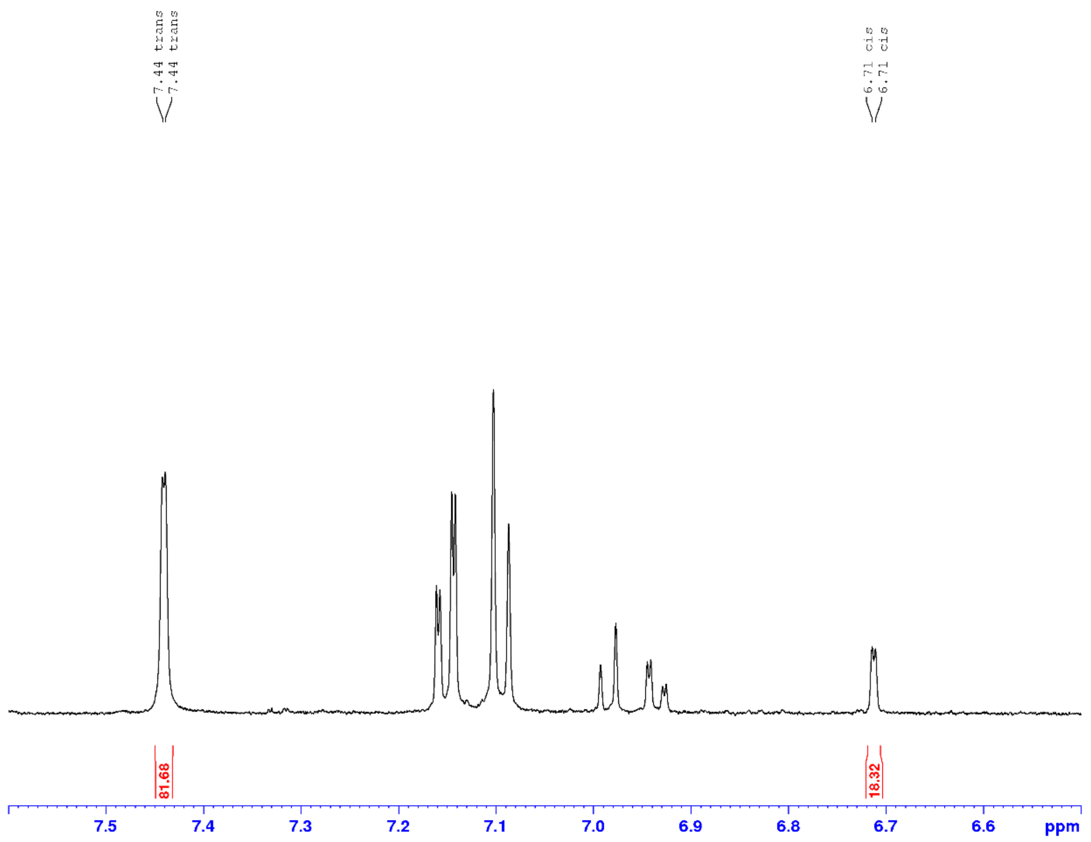


**Figure S36**: $^{1}$H-NMR spectrum of compound **5b** at PSS (385 nm).

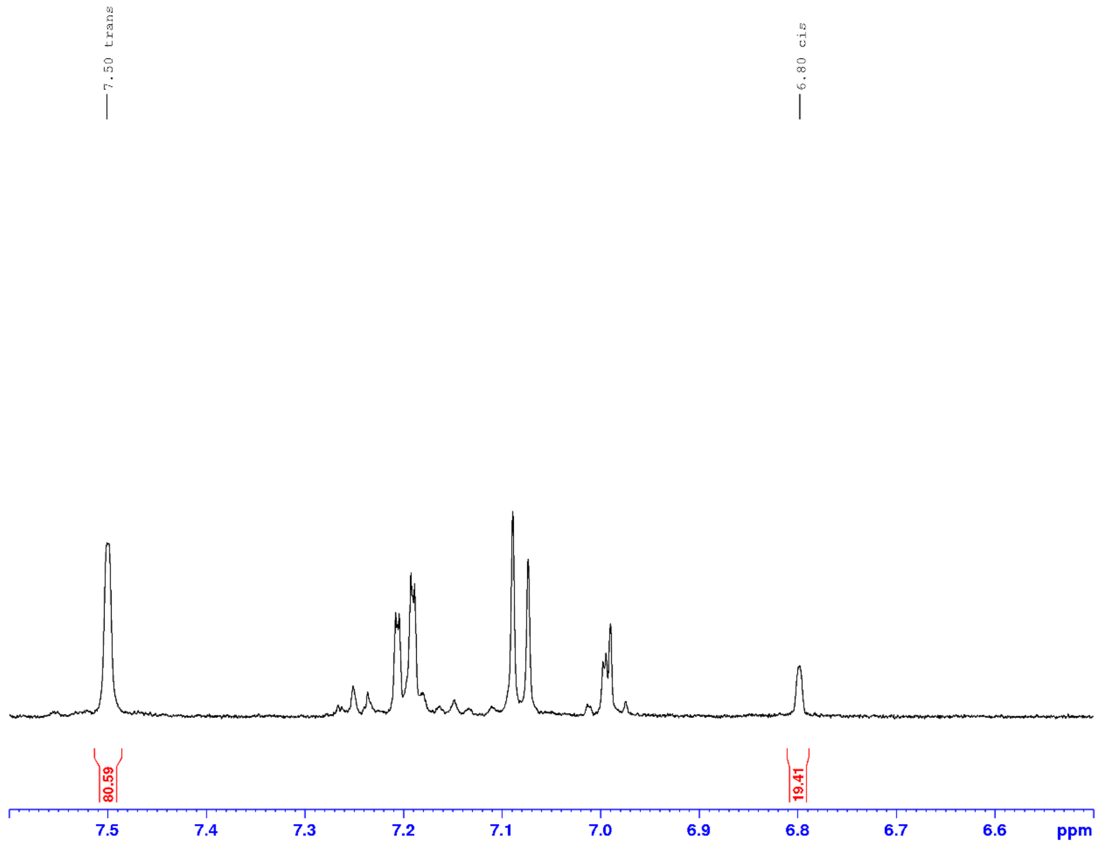


**Figure S37**: $^{1}$H-NMR spectrum of compound **6a** at PSS (385 nm).

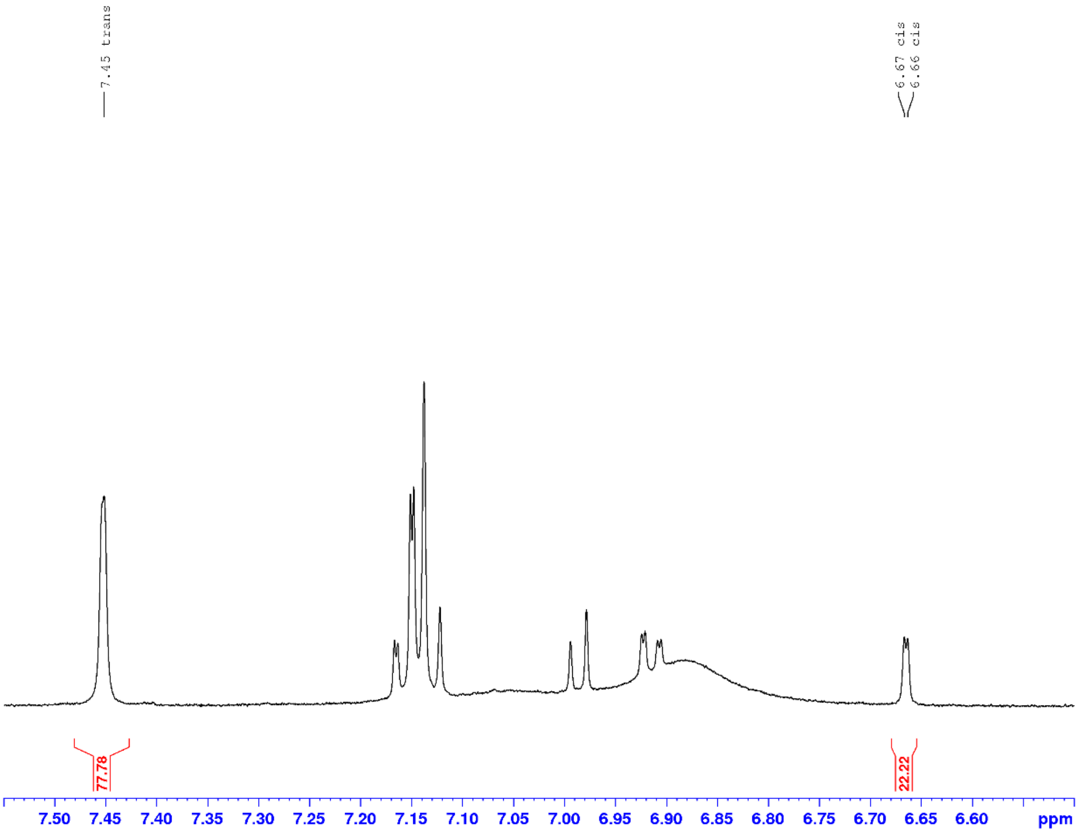


**Figure S38**: $^{1}$H-NMR spectrum of compound **6b** at PSS (385 nm).

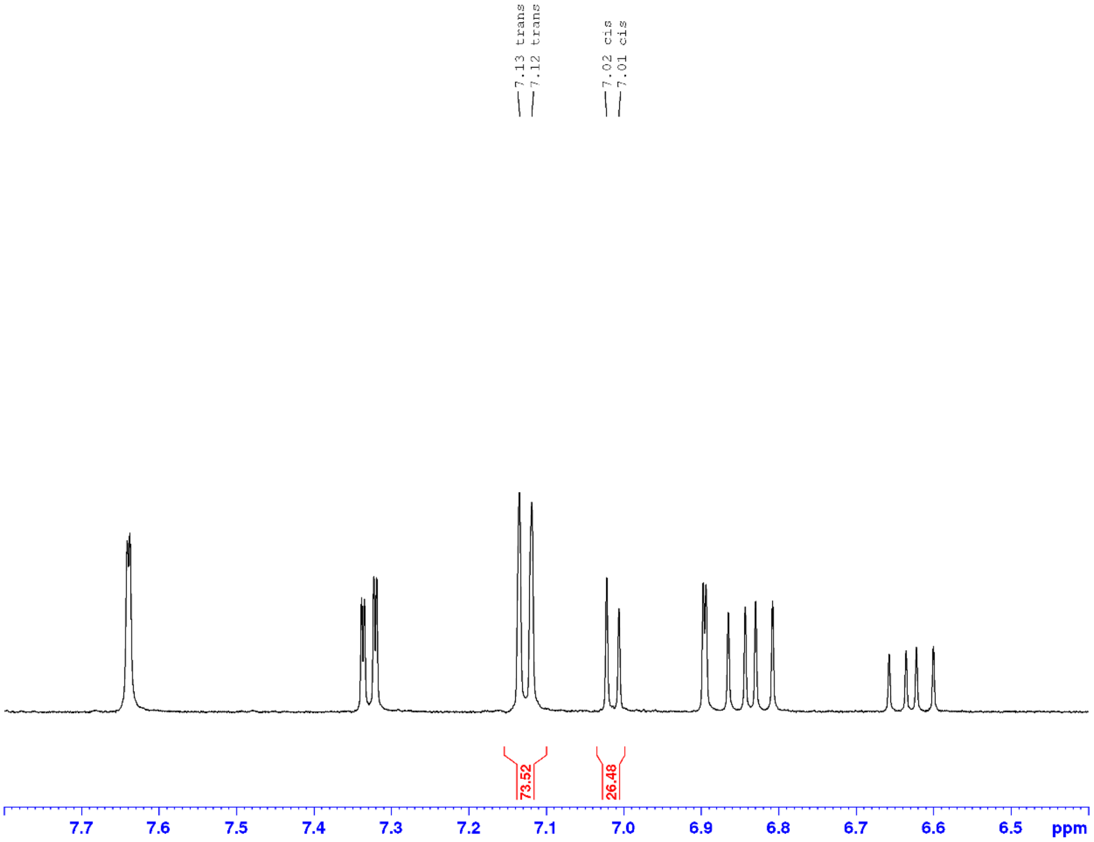


**Figure S39**: $^{1}$H-NMR spectrum of compound **7** at PSS (385 nm).

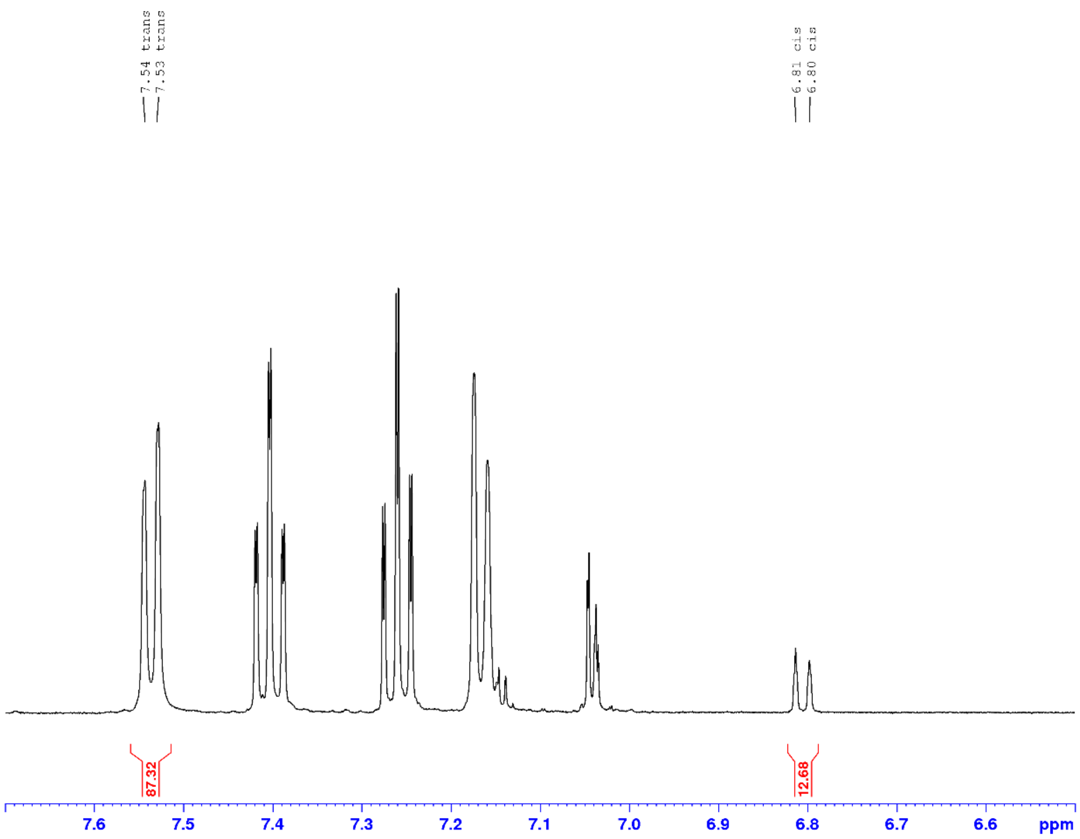


**Figure S40**: $^{1}$H-NMR spectrum of compound **1** at PSS (385 nm).

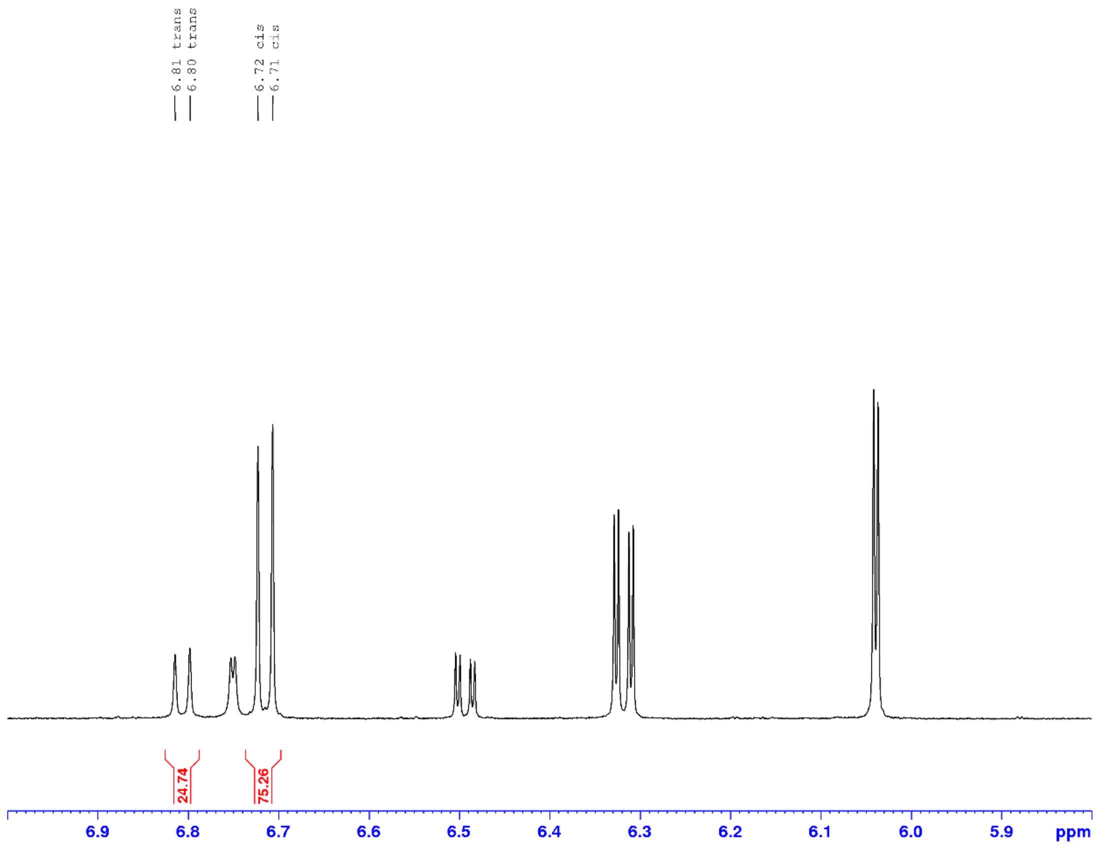


**Figure S41**: $^{1}$H-NMR spectrum of compound **3** at PSS (385 nm).

## Thermal half-lives determined by UV–vis spectroscopy

The functionalized diazocines **1–7**,**11a** and **11b** were dissolved in acetonitrile under the exclusion of light and UV–vis *cis* spectra were recorded at 298.15 K. Then the solutions were irradiated at 385 nm for 2 min. Spectra were recorded in appropriate intervals until complete relaxation. The absorption maxima were plotted as a function of time and the reaction time constants were determined after exponential fitting.

**Table S1:** Thermal half-lives (298.15 K), determined by UV–vis spectroscopy in acetonitrile.

| **molecule** | **concentration [mM]** | **$t_{1/2}$(UV) [h] at 298.15 K** | **standard error [h]** |
|---|---|---|---|
| **1** | 2.0 | 15.3 | 0.32 |
| **2** | 1.3 | 24.8 | 0.57 |
| **3** | 1.3 | 20 | 0.80 |
| **4a** | 1.5 | 11.4 | 0.01 |
| **4b** | 1.5 | 16.7 | 0.87 |
| **5a** | 1.0 | 11.2 | 0.22 |
| **5b** | 1.7 | 14.0 | 1.10 |
| **6a** | 1.2 | 14.7 | 0.29 |
| **6b** | 1.6 | 10.1 | 0.47 |
| **7** | 1.6 | 13.1 | 1.03 |
| **11a** | 1.5 | 10.2 | 0.01 |
| **11b** | 1.5 | 15.9 | 0.36 |
